# Protein embeddings improve phage-host interaction prediction

**DOI:** 10.1371/journal.pone.0289030

**Published:** 2023-07-24

**Authors:** Mark Edward M. Gonzales, Jennifer C. Ureta, Anish M. S. Shrestha

**Affiliations:** 1 Bioinformatics Laboratory, Advanced Research Institute for Informatics, Computing and Networking, De La Salle University, Manila, Philippines; 2 Systems and Computational Biology Research Unit, Center for Natural Sciences and Environmental Research, De La Salle University, Manila, Philippines; 3 Department of Software Technology, College of Computer Studies, De La Salle University, Manila, Philippines; Nitte University, INDIA

## Abstract

With the growing interest in using phages to combat antimicrobial resistance, computational methods for predicting phage-host interactions have been explored to help shortlist candidate phages. Most existing models consider entire proteomes and rely on manual feature engineering, which poses difficulty in selecting the most informative sequence properties to serve as input to the model. In this paper, we framed phage-host interaction prediction as a multiclass classification problem that takes as input the embeddings of a phage’s receptor-binding proteins, which are known to be the key machinery for host recognition, and predicts the host genus. We explored different protein language models to automatically encode these protein sequences into dense embeddings without the need for additional alignment or structural information. We show that the use of embeddings of receptor-binding proteins presents improvements over handcrafted genomic and protein sequence features. The highest performance was obtained using the transformer-based protein language model ProtT5, resulting in a 3% to 4% increase in weighted F1 and recall scores across different prediction confidence thresholds, compared to using selected handcrafted sequence features.

## Introduction

One of the most pressing threats to global health is antimicrobial resistance (AMR), a phenomenon wherein microorganisms evolve to withstand exposure to bacteriostatic and bactericidal drugs. In 2019, 4.95 million AMR-related and 1.27 million AMR-attributable deaths were estimated [1]. In developing countries, this problem is compounded by the unregulated dispensation of antibiotics as a form of self-medication even for mild conditions [2, 3] and their routine use in the agricultural sector for disease prophylaxis [4] and livestock growth promotion [5].

A solution that is actively being explored to combat this problem is phage therapy, which capitalizes on the specificity of bacteriophages (hereinafter referred to as *phages*) to a narrow range of hosts. Phages have been shown to antagonize the target bacteria with minimal side effects and without triggering a dysbiosis of the beneficial microbiota [6]. However, the foremost challenge to formulating phage cocktails for treating bacterial infections is identifying putative phages that attack the offending pathogens. Aside from being time- and cost-intensive, *in vitro* experiments require the cultivation of microbes under strict laboratory conditions, posing a bottleneck to the rapid selection of candidate phages.

With the advent of high-throughput sequencing technologies and the resulting increase in omic data, *in silico* approaches have been employed to help shortlist candidate phages. These can be broadly categorized into alignment-based methods [7, 8], which rely on sequence similarity to infer phage-host pairs, and alignment-free methods [9–11], which exploit features related to sequence composition, such as oligonucleotide frequency and codon usage bias. These reflect shared genomic properties that arise from the close coexistence and coevolution of phages and their hosts [12, 13].

Machine learning algorithms for phage-host interaction prediction have also been actively explored. Feature sets extracted from protein sequences include molecular weight [14], aromaticity [15], amino acid composition [14], protein-protein and domain-domain interaction [14, 16], and protein secondary structure [15]. Meanwhile, those obtained from genomic sequences include *k*-mer frequency [14, 15, 17], guanine-cytosine content [15], codon usage bias [15, 16], oligonucleotide frequency [16, 18], and shared transfer ribonucleic acids [19]. Recent studies have also investigated the application of deep learning architectures, primarily convolutional neural networks, that take these handcrafted properties as input [20–23].

While these existing models have been successful in integrating various features to improve their performance, most consider the entire proteome of both the phages and their hosts [14, 16, 18–21, 23], when only specific proteins are actually involved in phage-host interaction [24]. To initiate infection, a tailed phage typically adsorbs to the host bacterium’s surface through receptor-binding proteins (RBPs) located at its tail’s distal end [25]. In this regard, RBPs (e.g., tail fibers and spikes) serve as key machinery for host recognition and specificity [26–29].

Moreover, most tools for phage-host interaction prediction rely on manual feature engineering to transform raw sequences into numerical vectors, often requiring additional alignment or structural information [30–32]. The multitude of potentially informative signals that can be derived from these sequences also poses difficulty in selecting which features should be fed to the model [30].

Representation learning, in which raw biological sequences are converted into dense vectors in high-dimensional space, has recently been applied to some prototypical bioinformatics tasks, such as predicting protein function [33], succinylation sites [34], and sequence conservation [35]. The embeddings produced by protein language models, such as SeqVec [36] (which adopts the architecture of the natural language model ELMo [37]) and the transformer-based Evolutionary Scale Modeling (ESM) [38] and ProtTrans [31], have been demonstrated to capture protein secondary structure and physicochemical characteristics, features that are relevant to phage-host specificity [31, 38]. Representation learning also serves as a promising approach to address some of the limitations posed by the problem of data scarcity in phage-host datasets [13], as knowledge is distilled from large-scale protein databases [39–42]. However, the application of protein embeddings to the problem of phage-host interaction prediction remains unexplored.

In an attempt to address these gaps, our study seeks to contribute the following:

- We framed phage-host interaction prediction as a multiclass classification problem, with the embedding of a receptor-binding protein (RBP) as the input and the host genus as the output.
- We extensively tested different protein language models to automatically generate dense embeddings of RBP sequences.
- We constructed a random forest model for predicting phage-host interaction and showed that embeddings of RBPs outperform handcrafted genomic and protein sequence features, with the use of the protein language model ProtT5 resulting in the highest performance.

## Materials and methods

**Fig 1:**
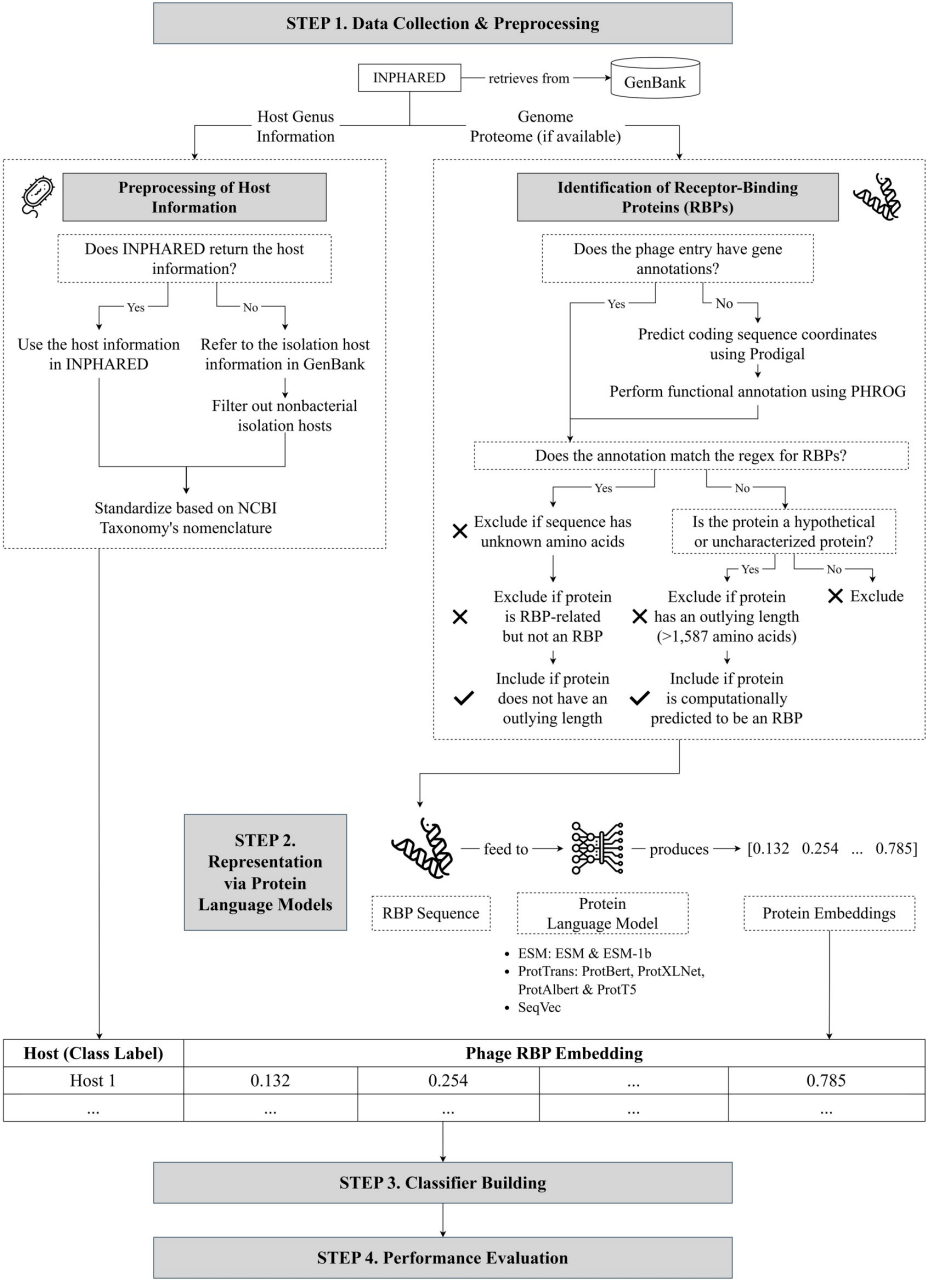
Methodology of our study. Step 1: We collected phage genomes, along with their proteomes (see Section Data collection and preprocessing for more details), and performed preprocessing to obtain the host information (see Section Preprocessing of host information) and select annotated receptor-binding proteins or RBPs (see Section Identification of receptor-binding proteins). Step 2: We fed the RBP sequences to pretrained protein language models to generate meaningful dense embeddings (see Section Representation via protein language models). Step 3: We built a random forest model with the RBP embeddings as the input and the host genus as the predicted output (see Section Classifier building). Step 4: We evaluated our model’s performance (see Section Performance evaluation). Flat icons used in this figure are taken from [43–45].

In this section, we discuss the methodology of our study (Fig 1). For the purpose of reproducibility, the data and source code for all our experiments and analysis are publicly available at https://github.com/bioinfodlsu/phage-host-prediction. Detailed instructions on setting up the environment, installing the required dependencies, and running the code are also provided in this repository.

### Data collection and preprocessing

We collected genome sequences of 18,389 phages, along with their proteome sequences (whenever available), via INPHARED [46], a pipeline for retrieving phage sequences from GenBank [47]; the sequences were downloaded in September 2022, and proteomes were retrieved for 16,836 phages. Limiting the host information to the genus level, we subjected the entries to preprocessing of host information and selection of annotated receptor-binding proteins, which we describe in detail in the following subsections.

#### Preprocessing of host information

INPHARED [46] returns host data for 15,739 phages across 278 different host genera. For entries where the host name is unspecified, we referred to the isolation host information in GenBank whenever available and filtered out nonbacterial isolation hosts. We used Biopython [48], an open-source suite of bioinformatics tools written in Python, in parsing the phages’ GenBank records. We then standardized the host names following NCBI Taxonomy’s nomenclature [49]. Note that, while some phages are known to be polyvalent (multihost), only five phage entries were recorded with multiple host genera. Hence, for simplicity, we mapped each polyvalent phage to its host with the highest number of interacting phages in the dataset.

After preprocessing, host information was supplied for an additional 84 phages, thus totaling 15,823 phages across 279 host genera; the additional identified genus, *Silvanigrella*, was from the isolation host of MWH-Nonnen-W8red.

#### Identification of receptor-binding proteins

Among the phage entries with host data, 15,158 entries have gene annotations, while the remaining 665 do not. For those with annotations, we selected the annotated receptor-binding proteins (RBPs) using a regular expression (S1 Listing in S1 File) and a manual exclusion list adapted from Boeckaerts *et al*. [50]; this exclusion list covers proteins related to RBPs but are not RBPs themselves (e.g., assembly and portal proteins). We also discarded sequences with undetermined amino acids (X).

Meanwhile, we ran the genomes of phages without annotation through Prokka [51], a wrapper tool for genome annotation. It first calls Prodigal [52] to predict the coding sequence coordinates. To identify the putative gene products, we configured Prokka [51] to refer to PHROG [53], a database of viral protein family clusters generated by employing hidden Markov model profile-profile comparisons for remote homology detection. PHROG [53] has also been used in previous studies that require the functional annotation of phage protein sequences [54, 55]. The annotated RBPs were selected following the same scheme described in the previous paragraph.

**Fig 2:**
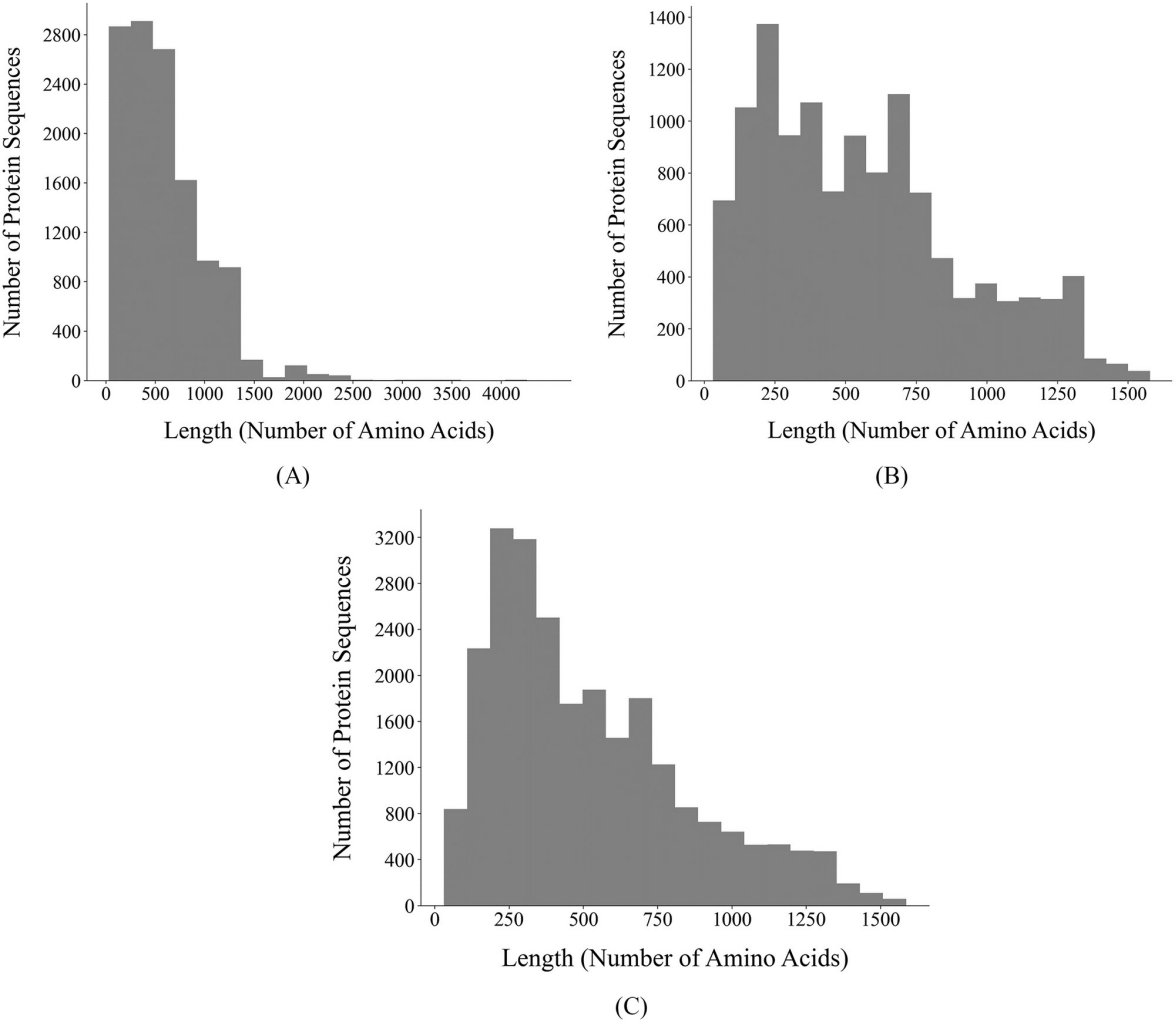
Distribution of the lengths of the receptor-binding proteins (RBPs). (A) Distribution of the lengths of the annotated RBPs selected based on annotation in GenBank and the functional annotation obtained using PHROG [53]. (B) Distribution of the lengths of the annotated RBPs after excluding those longer than 1,587 amino acids. This cutoff was set by defining outlying lengths as those outside the interval [*Q*_1_ − 1.5 ⋅ *IQR*, *Q*_3_ + 1.5 ⋅ *IQR*], where *Q*_1_ is the first quartile, *Q*_3_ is the third quartile, and *IQR* is the interquartile range of the RBP lengths. (C) Distribution of the lengths of all the RBPs in our dataset, including those computationally predicted via the approach proposed by Boeckaerts *et al*. [50].

Afterwards, we discarded RBPs with lengths outside the interval [*Q*_1_ − 1.5 ⋅ *IQR*, *Q*_3_ + 1.5 ⋅ *IQR*], where *Q*_1_ is the first quartile, *Q*_3_ is the third quartile, and *IQR* is the interquartile range of the RBP lengths. This resulted in the removal of protein sequences longer than 1,587 amino acids. Fig 2A and 2B show the distribution of the lengths of the RBPs before and after this step, respectively.

Finally, to expand the list of RBPs in our dataset, we also considered proteins labeled as hypothetical by GenBank or uncharacterized by Prokka. Following Boeckaerts *et al*. [50], we encoded these sequences via the transformer-based protein language model ProtBert [31] and fed the generated embeddings to their proposed extreme gradient boosting model to computationally predict whether the hypothetical proteins are RBPs or not. In total, our dataset consists of 24,752 RBPs across 9,583 phages and 232 hosts (Table 1); the distribution of the lengths of these RBPs is presented in Fig 2C.

**Table 1:** Statistics on the identification of receptor-binding proteins (RBPs). GenBank refers to the RBPs annotated in GenBank, PHROG refers to those selected based on the functional annotation obtained using PHROG [53]. *Predicted* refers to those computationally predicted via the approach proposed by Boeckaerts *et al*. [50].

	Num. of RBPs	Num. of Phages	Num. of Hosts
GenBank	11,133	5,805	167
PHROG	1,010	419	30
Predicted	12,609	5,941	192
Total	24,752	9,583	232

### Representation via protein language models

To generate dense vector representations (embeddings) of the RBP sequences, we explored seven pretrained protein language models as feature encoders: ESM [38], ESM-1b [38], ProtBert [31], ProtXLNet [31], ProtAlbert [31], ProtT5 [31], and SeqVec [36]. To this end, we used bio_embeddings [56], an open-source Python library that provides reproducible workflows for applying representation learning to protein sequences. These protein language models adopt deep learning architectures and are pretrained on large-scale protein databases with the goal of capturing biophysical features that may be relevant to downstream tasks.

SeqVec [36] adopts ELMo [37], which consists of a character-level convolutional neural network and two bidirectional long short-term memory networks. ESM1-b [38] is a 33-layer transformer built by optimizing the hyperparameters of the 34-layer transformer ESM. [38]. ProtBert [31], ProtAlbert [31], and ProtT5 [31] are 30-layer, 12-layer, and 24-layer autoencoding transformers, respectively. ProtXLNet [31] is a 30-layer autoregressive transformer. Additional technical details about these protein language models are provided in Table 2.

**Table 2:** Protein language models for generating receptor-binding protein embeddings.

Model	Architecture	Pretraining Datasets	Vector length
SeqVec [36]	ELMo [37]	UniRef50 [40]	1024
ESM [38]	Transformer	UniParc [39]	1280
ESM-1b [38]	Transformer	UniRef50 [40]	1280
ProtBert [31]	Transformer	UniRef100 [40], BFD100 [41, 42]	1024
ProtXLNet [31]	Transformer	UniRef100 [40]	1024
ProtAlbert [31]	Transformer	UniRef100 [40]	4096
ProtT5 [31]	Transformer	UniRef50 [40], BFD100 [41, 42]	1024

Since these protein language models output embeddings per “token” (i.e., per residue), we performed averaging over the residues to produce fixed-length protein embeddings [31, 36, 38]. Formally, suppose a protein sequence with *r* residues is fed as input to a protein language model that encodes each residue as an embedding of length *s*. The output of this model is an *r* × *s* matrix **M** whose *i*^th^ row is the embedding of the *i*^th^ residue. From this matrix **M**, we obtain the protein embedding of length *s*, which we denote by **v**, via Eq (1).

$$\begin{array}{c} \text{v}=[\begin{array}{ccccc} \frac{1}{r}\sum_{i=1}^{r}{\text{M}}_{i1}, & \frac{1}{r}\sum_{i=1}^{r}{\text{M}}_{i2}, & \frac{1}{r}\sum_{i=1}^{r}{\text{M}}_{i3}, & \cdots , & \frac{1}{r}\sum_{i=1}^{r}{\text{M}}_{is} \end{array}] \end{array}$$ (1)

Note that, for ESM and ESM-1b (which accept sequences with at most 1022 residues only), we followed the approach of Marquet *et al*. [35] and split sequences longer than 1022 residues into non-overlapping subsequences of length 1022 (whenever possible). We fed each subsequence to the protein language model separately, resulting in a set of matrices corresponding to the per-residue embeddings. We then stacked these matrices into an *r* × *s* matrix **M** as described in the previous paragraph, and applied Eq (1) to obtain the protein embedding.

### Classifier building

We framed phage-host interaction prediction as a multiclass classification problem, with the protein embeddings of the RBPs as the input and the host (at the genus level) as the output. Including all 232 hosts in our dataset resulted in class imbalance, with a quarter of the hosts already accounting for 96.02% of the dataset entries. To mitigate this, we restricted the class labels to only the top 25% (i.e., 58) hosts associated with the most RBPs. The class labels are enumerated in S1 Table in S1 File.

We divided our dataset into two sets *D*_1_ and *D*_2_, where *D*_1_ contains the RBPs with class labels belonging to the top 25% hosts and *D*_2_ contains the remaining entries. We then partitioned *D*_1_ following a stratified 70%-30% train-test split and appended *D*_2_ to the test set. As such, our test set includes RBPs with class labels outside the top 25% hosts, which we will refer to as the *others* class. This is to make the evaluation more reflective of real-world use cases, where we might encounter inputs not belonging to any class for which the model was trained. In total, our training and test sets have 16,636 and 8,116 samples, respectively. Table 3 reports the training and test set statistics on the top 10 hosts associated with the most RBPs; the complete statistics are given in S1 Table in S1 File.

**Table 3:** Number of training and test samples for the top 10 hosts associated with the most receptor-binding proteins (RBPs). The RBPs associated with the top 10 hosts comprise 64.66% of our dataset. Four of the top 10 hosts (*Escherichia*, *Salmonella*, *Klebsiella*, and *Erwinia*) belong to the same order: Enterobacterales.

Host	Training Set	Test Set	Total
*Escherichia*	3,021	1,295	4,316
*Salmonella*	1,474	632	2,106
*Synechococcus*	1,216	521	1,737
*Pseudomonas*	1,196	513	1,709
*Vibrio*	1079	463	1,542
*Klebsiella*	926	397	1,323
*Erwinia*	667	286	953
*Mycobacterium*	578	248	826
*Staphylococcus*	568	244	812
*Bacillus*	475	204	679

Afterwards, we fed the protein embeddings to a random forest classifier built using scikit-learn [57], an open-source Python library for machine learning. In a further attempt to address class imbalance, we employed a weighted random forest model that imposes higher misclassification penalties for minority classes [58, 59]. Formally, let *T* be the total number of samples in the training set, *C* be the set of class labels, and *t*_*c*_ be the number of training samples under class *c* ∈ *C*. The misclassification penalty *w*_*c*_ for class *c* is given by Eq (2).

$$\begin{array}{c} w_{c}=\frac{T}{t_{c}\cdot \left|C\right|} \end{array}$$ (2)

To optimize the weighted F1 score, we conducted hyperparameter tuning with five-fold stratified cross-validation. The hyperparameter space is as follows (the optimal values are in bold): number of trees (50, 100, **150**, 200), number of features to consider in determining the best split (log_2_, **square root**), minimum number of samples to split an internal node (**2**, 3, 4), and minimum number of samples to be a leaf node (**1**, 2, 3, 4).

### Performance evaluation

In order to factor in our model’s confidence in its prediction, we introduced a confidence threshold *k*. Let *p*_1_ and *p*_2_ be the highest and second-highest predicted class probabilities, respectively, for an input RBP. This input is classified under its predicted class label if and only if *p*_1_ − *p*_2_ ≥ *k*. If *p*_1_ − *p*_2_ < *k*, then it is classified as *others* since the model is ambiguous about its classification. S2 Table in S1 File defines the true and false positive and negative outcomes in view of this scheme.

We evaluated our model’s performance using weighted precision, recall, F1, and specificity, with the weights corresponding to the class sizes. Formally, let *N* be the total number of samples in the test set, *C* be the set of class labels including the *others* class, and *n*_*c*_ be the number of test samples under class *c* ∈ *C*. Let *TP*_*c*,*k*_, *TN*_*c*,*k*_, *FP*_*c*,*k*_, and *FN*_*c*,*k*_ be the number of true positive, true negative, false positive, and false negative outcomes for class *c* at confidence threshold *k*. The definitions of the evaluation metrics are given in Eqs (3) to (5).

$$\begin{array}{c} {\text{Weighted-Precision}}_{k}=\frac{1}{N}\sum_{c\in C}n_{c}\cdot \frac{TP_{c,k}}{TP_{c,k}+FP_{c,k}} \end{array}$$ (3)

$$\begin{array}{c} {\text{Weighted-Recall}}_{k}=\frac{1}{N}\sum_{c\in C}n_{c}\cdot \frac{TP_{c,k}}{TP_{c,k}+FN_{c,k}} \end{array}$$ (4)

$$\begin{array}{c} {\text{Weighted-Specificity}}_{k}=\frac{1}{N}\sum_{c\in C}n_{c}\cdot \frac{TN_{c,k}}{TN_{c,k}+FP_{c,k}} \end{array}$$ (5)

$$\begin{array}{c} {\text{Weighted-F1}}_{k}=\frac{1}{N}\sum_{c\in C}n_{c}\cdot \frac{2\cdot \frac{TP_{c,k}}{TP_{c,k}+FP_{c,k}}\cdot \frac{TP_{c,k}}{TP_{c,k}+FN_{c,k}}}{\frac{TP_{c,k}}{TP_{c,k}+FP_{c,k}}+\frac{TP_{c,k}}{TP_{c,k}+FN_{c,k}}} \end{array}$$ (6)

## Results

### Transformer-based embeddings of receptor-binding proteins outperformed handcrafted genomic and protein features for phage-host interaction prediction

We compared the performance of our model with that of the state-of-the-art phage-host interaction prediction tool by Boeckaerts *et al*. [15]; its feature set consists of 218 handcrafted features, 133 of which are derived from the genomic sequences and 85 from the RBP sequences. To ensure a fair comparison, we retrained this tool on our training dataset. We evaluated the performance across different confidence thresholds ranging from *k* = 60% to 100% in steps of 10%.

While representing RBPs via protein embeddings and via these handcrafted features yielded similar weighted precision and specificity scores (S3, S5 Tables in S1 File), the use of protein embeddings improved the weighted F1 and recall across all tested confidence thresholds (Table 4 and S4 Table in S1 File). Among the evaluated protein language models, the highest performance was obtained with the autoencoder model ProtT5, followed by ESM-1b and ESM. In particular, utilizing the ProtT5 embeddings outperformed the handcrafted features by around 3% to 4% in terms of weighted F1 and recall. Meanwhile, the smallest performance increase was registered by the autoregressive model ProtXLNet. The per-class evaluation results are reported in S6 to S10 Tables in S1 File.

**Table 4:** Model performance in terms of weighted F1. The header row refers to the confidence thresholds at which we evaluated model performance; these confidence thresholds range from *k* = 60% to 100% in steps of 10%.

	60%	70%	80%	90%	100%
Boeckaerts *et al*. [15]	59.35%	53.41%	47.20%	38.97%	21.61%
SeqVec	60.64%	54.90%	49.12%	40.52%	23.20%
ESM	62.21%	57.13%	51.18%	42.70%	**25.18%**
ESM-1b	62.27%	57.16%	51.29%	42.66%	25.04%
ProtBert	60.58%	55.27%	49.29%	41.07%	24.59%
ProtXLNet	60.18%	54.54%	48.46%	40.20%	23.92%
ProtAlbert	60.45%	54.82%	48.51%	40.57%	23.80%
ProtT5	**62.95%**	**57.51%**	**51.98%**	**43.05%**	24.82%

The highest weighted F1 scores are given in bold and underlined.

**Fig 3:**
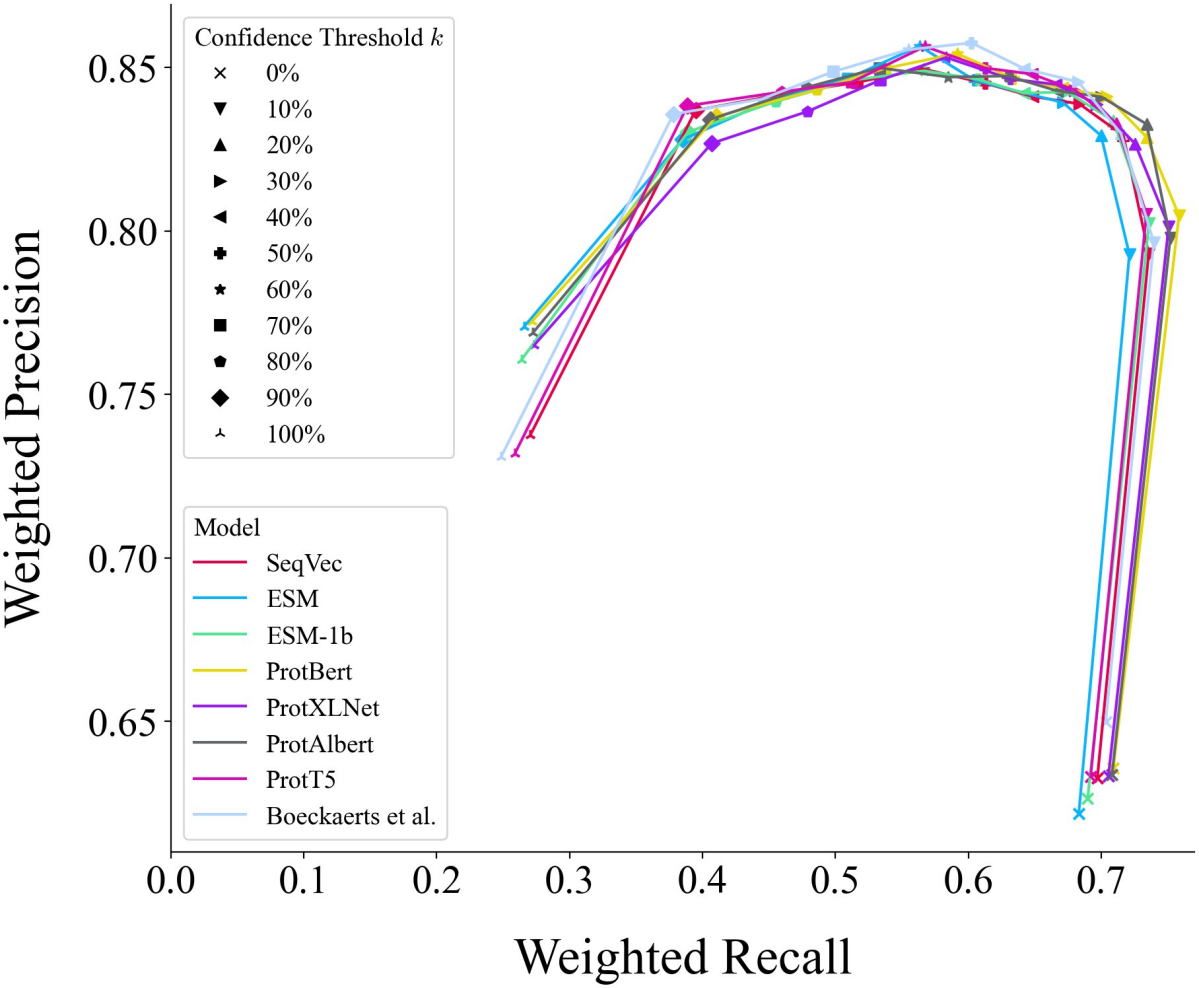
Weighted precision-recall curves showing the model performance. The curves plot the weighted precision against the weighted recall at different confidence thresholds ranging from *k* = 0% to 100% in steps of 10%.

Fig 3 plots the weighted precision against the weighted recall scores at confidence thresholds ranging from *k* = 0% to 100% in steps of 10%. From *k* = 10% to around *k* = 50% or 60%, increasing the value of *k* increases the precision but decreases the recall. Beyond this confidence threshold, increasing the value of *k* decreases both precision and recall. A reason for this decrease in precision is that setting *k* to higher (stricter) values results in more samples being mislabeled under the *others* class, which comprises around 12% of our test set. We also provide the weighted precision-recall curve at *k* = 0 (i.e., when the confidence measure is removed and samples are classified according highest class probability resulting in none of the samples, including truly others, as being labeled as *others*) in S1 Fig in S1 File.

### Integrating handcrafted features did not significantly increase performance

To determine the extent to which the further integration of handcrafted sequence properties can potentially improve the predictive power of our best-performing model, we combined the ProtT5 embeddings of the RBPs with the sequence properties that registered the highest Gini importance after training the model by Boeckaerts *et al*. [15] on our dataset. We also examined the performance if the sequence properties were limited to those extracted from protein sequences.

As reported in Tables 5 to 8, the increase in the weighted F1 scores after integrating these handcrafted sequence properties was consistently below 1.33% across all tested confidence thresholds. Furthermore, the increase in precision, recall, and specificity was also consistently below 1.40% (S11 to S14 Tables in S1 File), 1.41% (S15 to S18 Tables in S1 File), respectively, and 0.18% (S19 to S22 Tables in S1 File). Along with the weighted precision-recall curves in S2 to S5 Figs in S1 File, these results suggest that the ProtT5 embeddings may already be capturing these sequence properties.

**Table 5:** Weighted F1 scores after integrating handcrafted sequence properties to the vector representations of the receptor-binding proteins. The selected sequence properties are those with the highest Gini importance after training the phage-host interaction prediction tool by Boeckaerts *et al*. [15] on our dataset. The header row refers to the confidence thresholds at which we evaluated model performance.

	60%	70%	80%	90%	100%
ProtT5	62.95%	57.51%	51.98%	43.05%	24.82%
ProtT5 + A Nucleotide Frequency	63.49%	58.36%	52.56%	43.37%	24.93%
ProtT5 + GC Content	63.91%	58.60%	52.60%	43.99%	25.57%
ProtT5 + C Nucleotide Frequency	63.57%	58.47%	52.44%	43.48%	25.09%
ProtT5 + TTA Codon Frequency	63.25%	57.60%	51.75%	43.01%	24.97%
ProtT5 + TTA Codon Usage Bias	63.22%	57.56%	51.70%	43.05%	24.63%

**Table 6:** Weighted F1 scores after integrating handcrafted protein sequence properties to the vector representations of the receptor-binding proteins. The selected protein sequence properties are those with the highest Gini importance after training the phage-host interaction prediction tool by Boeckaerts *et al*. [15] on our dataset. The header row refers to the confidence thresholds at which we evaluated model performance.

	60%	70%	80%	90%	100%
ProtT5	62.95%	57.51%	51.98%	43.05%	24.82%
ProtT5 + K (Lysine) Frequency	63.00%	57.49%	52.00%	43.13%	25.07%
ProtT5 + Isoelectric Point (pI)	62.90%	57.36%	51.76%	43.01%	25.27%
ProtT5 + Fourth Protein Z-Scale*	62.86%	57.40%	51.48%	43.06%	24.97%
ProtT5 + % of Exposed SA^†^	62.78%	57.65%	51.64%	42.91%	25.42%
ProtT5 + Molecular Weight	62.98%	57.74%	51.79%	42.87%	25.15%

* The fourth protein Z-scale (Z4) [60] is related to the heat of formation, hardness, electronegativity, and electrophilicity.

^†^ *% of Exposed SA* refers to the percentage of residues with exposed solvent accessibility.

**Table 7:** Weighted F1 scores after integrating the top *n* handcrafted sequence properties to the vector representations of the receptor-binding proteins. The selected sequence properties are those with the highest Gini importance after training the phage-host interaction prediction tool by Boeckaerts *et al*. [15] on our dataset. These properties (in order of decreasing importance) are the A nucleotide frequency, GC content, C nucleotide frequency, TTA codon frequency, and TTA codon usage bias. The header row refers to the confidence thresholds at which we evaluated model performance.

	60%	70%	80%	90%	100%
ProtT5	62.95%	57.51%	51.98%	43.05%	24.82%
ProtT5 + Top 1	63.49%	58.36%	52.56%	43.37%	24.93%
ProtT5 + Top 2	64.26%	58.76%	52.70%	43.75%	25.13%
ProtT5 + Top 3	64.17%	58.83%	52.81%	44.20%	25.33%
ProtT5 + Top 4	64.03%	58.83%	52.67%	43.85%	24.44%
ProtT5 + Top 5	64.01%	58.68%	52.77%	44.21%	24.80%

**Table 8:** Weighted F1 scores after integrating the top *n* handcrafted protein sequence properties to the vector representations of the receptor-binding proteins. The selected protein sequence properties are those with the highest Gini importance after training the phage-host interaction prediction tool by Boeckaerts *et al*. [15] on our dataset. These properties (in order of decreasing importance) are the K (lysine) frequency, isoelectric point, fourth protein Z-scale [60] (which is related to the heat of formation, hardness, electronegativity, and electrophilicity), percentage of residues with exposed solvent accessibility, and molecular weight. The header row refers to the confidence thresholds at which we evaluated model performance.

	60%	70%	80%	90%	100%
ProtT5	62.95%	57.51%	51.98%	43.05%	24.82%
ProtT5 + Protein Top 1	63.00%	57.49%	52.00%	43.13%	25.07%
ProtT5 + Protein Top 2	62.96%	57.57%	51.79%	42.87%	25.17%
ProtT5 + Protein Top 3	62.95%	57.47%	51.38%	43.09%	25.19%
ProtT5 + Protein Top 4	62.92%	57.63%	51.67%	43.36%	25.34%
ProtT5 + Protein Top 5	62.79%	57.82%	51.83%	42.86%	24.80%

## Discussion

### Representing receptor-binding proteins using protein language models

The main novelty of our study lies in our use of protein language models to obtain dense vector encodings of receptor-binding proteins, which are known to be major determinants of phage-host specificity [26–29].

Vectorizing sequences by representation learning requires only the sequences themselves, discarding the need to derive additional alignment or structural information and eliminating the difficulty of selecting from a wide array of potentially informative signals [30]. Aside from these general advantages of representation learning over manual feature engineering, our experiments also showed that the use of protein embeddings improves phage-host interaction prediction and outperforms handcrafted genomic and protein features. In particular, utilizing the transformer-based autoencoder model ProtT5 resulted in the best performance, increasing the weighted F1 and recall scores by 3% to 4% across all tested confidence thresholds.

### Our embeddings-based model captures a complex combination of features

While protein embeddings have been shown to improve performance in several prototypical bioinformatics tasks [34, 61, 62], their interpretability remains a challenge [63]. The most common approach is to project the embeddings onto a low-dimensional space via nonlinear dimensionality reduction techniques [30, 31, 36, 64], such as *t*-distributed stochastic neighbor embedding (*t*-SNE) [65] and uniform manifold approximation and projection (UMAP) [66], and check for the presence of formed clusters.

These approaches have been employed to establish that protein embeddings carry information on salient physicochemical, structural, and functional properties of protein sequences [33]. For instance, SeqVec and the ProtTrans and ESM families of language models, which we explored in our work, have been shown to capture properties such as hydrophobicity, charge, polarity, and molecular weight [31, 36, 38]. In our experiments, we found no significant performance improvement after combining the ProtT5 embeddings with selected handcrafted features, suggesting that the embeddings may already be capturing guanine-cytosine content [15, 67–69], codon usage bias [15, 70–72], and other important signals of phage-host interaction that emerge from the close coexistence and coevolution of phages and their bacterial hosts.

**Fig 4:**
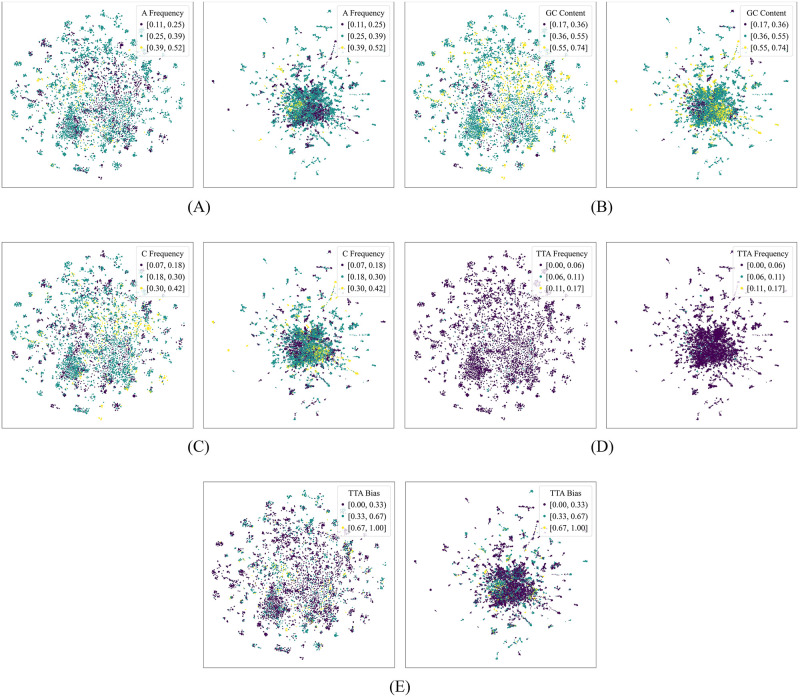
*t*-distributed stochastic neighbor embedding (*t*-SNE) and uniform manifold approximation and projection (UMAP) plots of the ProtT5 embeddings, colored based on handcrafted sequence properties. This figure shows the *t*-SNE (left of each subfigure) and UMAP (right of each subfigure) projections. Each point corresponds to the two-dimensional projection of a subvector of a receptor-binding protein’s ProtT5 embedding, the components of which are the *ℓ* components with the highest Gini importance after training our phage-host interaction prediction model (in this figure, *ℓ* = 100). The points were colored based on the sequence properties with the highest Gini importance after training the model by Boeckaerts *et al*. [15] on our dataset; these properties are as follows: (A) A nucleotide frequency, (B) GC content, (C) C nucleotide frequency, (D) TTA codon frequency, and (E) TTA codon usage bias (TTA codes for the amino acid leucine).

**Fig 5:**
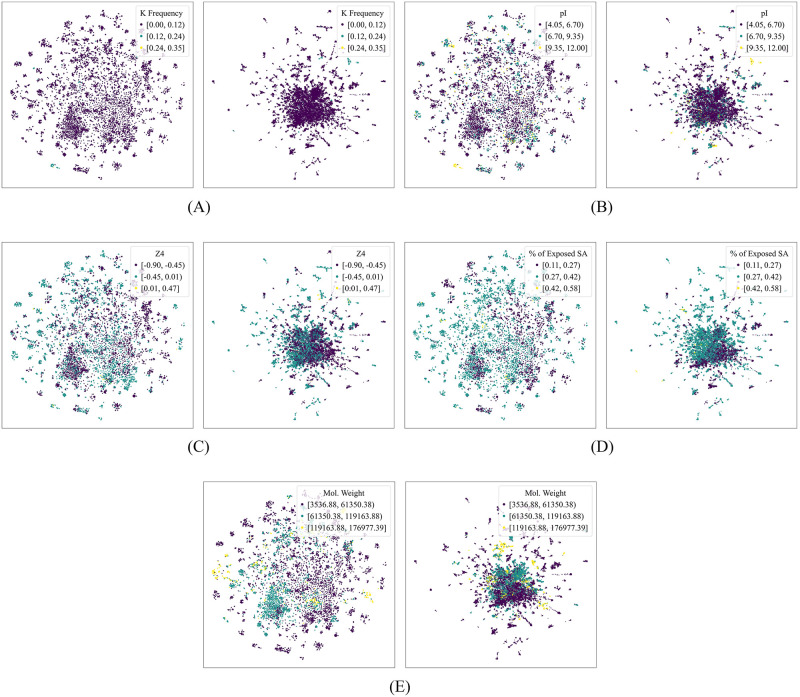
*t*-distributed stochastic neighbor embedding (*t*-SNE) and uniform manifold approximation and projection (UMAP) plots of the ProtT5 embeddings, colored based on handcrafted protein sequence properties. This figure shows the *t*-SNE (left of each subfigure) and UMAP (right of each subfigure) projections. Each point corresponds to the two-dimensional projection of a subvector of a receptor-binding protein’s ProtT5 embedding, the components of which are the *ℓ* components with the highest Gini importance after training our phage-host interaction prediction model (in this figure, *ℓ* = 100). The points were colored based on the protein sequence properties with the highest Gini importance after training the model by Boeckaerts *et al*. [15] on our dataset; these properties are as follows: (A) lysine frequency, (B) isoelectric point, (C) fourth protein Z-scale [60] (which is related to the heat of formation, hardness, electronegativity, and electrophilicity), (D) percentage of residues with exposed solvent accessibility, and (E) molecular weight.

To visualize the geometry of the embeddings and attempt to identify the biophysical features that they capture in the context of phage-host interaction prediction, we represented each RBP as a vector whose components are the ℓ components of its ProtT5 embedding with the highest Gini importance after training our model. We then employed *t*-SNE and UMAP [66] to project the set of these vectors onto a two-dimensional space. The *t*-SNE projections were generated by setting the perplexity to 50, the number of iterations to 1000, the initialization to principal component analysis, and the learning rate to the number of samples divided by 12 (following Kobak and Berens [73]). The UMAP projections were generated by setting the number of neighbors to 100 and the minimum distance between embedded points to 0.7. We colored the points based on the sequence properties with the highest Gini importance after training the model by Boeckaerts *et al*. [15] on our dataset. Figs 4 and 5 show the resulting plots when ℓ is set to 100.

Experimenting with different values of *ℓ*, we observed that, although there were instances wherein local clusters appeared to form, the points generally did not show clear separation boundaries, suggesting that the components of the embeddings do not squarely correspond to the individual sequence features under consideration.

Investigating the exact fashion in which these features are captured in the latent embedding space is a possible research direction, especially as improving the interpretability of deep learning models remains an open challenge. Vig *et al*. [74] viewed the attention weights across all the heads in a layer as a feature vector and fed this vector to classifiers designed for different probing tasks in order to determine whether the biophysical properties being probed are learned in the layer of interest. Another approach employed in previous studies [75, 76] is plotting heatmaps derived from the attention matrix to visualize and examine the attention weights. Recently, Hou *et al*. [77] used BertViz [78] to construct bipartite graphs that show the associations among the amino acids in the input sequence in view of self-attention. Utilizing other visualization tools from natural language processing, such as exBERT [79] and AttViz [80], is thus a promising direction that may be explored. Although it is not straightforward to infer the biophysical properties captured in the embeddings based only on these visualizations, they reveal key insights into the internal representations of sequences in transformer models, which, in turn, may serve as bases for biological hypotheses.

### Other formulations of the phage-host interaction prediction problem

Our study is primarily interested in investigating how the representation of phages’ receptor-binding proteins affects phage-host interaction prediction. To give emphasis on the representation of RBPs, we framed phage-host interaction prediction as a multiclass classification problem with the dense RBP embeddings as the input and the host genus as the output. We also benchmarked our work against a state-of-the-art method [15] that follows the same multiclass formulation but takes a different set of RBP properties (i.e., handcrafted genomic and protein features) as input.

A different formulation of the phage-host interaction prediction problem is as a binary classification problem, where the input is some representation of both phage and host sequences and the output is the presence (or absence) of interaction [14, 16, 20]. It can also be treated as a multiclass classification problem with some representation of the phage sequence as the input and the host as the output [17–19, 22, 23, 81]. Tools that follow this formulation typically have an under-the-hood dataset of information derived from calculating feature scores between phage sequences and a predetermined set of host sequences.

Moreover, while most studies [14, 16, 18–21, 23] consider the entire proteome or genome, our work and that of Boeckaerts *et al*. [15] narrow the input to some representation of selected phage proteins of biological interest (i.e., RBPs). Hence, instead of directly mapping the phages (given their genomes or proteomes) to putative hosts, we perform protein-to-host mapping, i.e., we map the RBPs to putative hosts of the phages from which these proteins were obtained.

While it is not possible to make a direct comparison of our results to the ones using a different approach than ours, we provide in Table 9 a summary of the computational problem formulation and performance of existing machine learning and deep learning tools for phage-host interaction prediction.

**Table 9:** Comparison of existing machine learning and deep learning tools for predicting phage-host interaction.

	Input	Host Level	Test Dataset*	Performance
Binary Classification (Entire Sequence as Input)				
Leite *et al*. [14]	Phage and host proteome	Strain	PhagesDB [82] + GenBank [47]	F1 = 95.9%
PHISDetector^†^ [16]	Phage and host genome	Species	VHM dataset [10]	Accuracy = 51%
PredPHI [20]	Phage and host proteome	Species	PhagesDB [82] + GenBank [47]	AUC-ROC = 81%
PHIAF [21]	Phage and host proteome and genome	Species	PhagesDB [82] + RefSeq [83] + MVP [84] + VHDB [85]	AUC-ROC = 88%
Multiclass Classification (Entire Sequence as Input)				
VirHostMatcher-Net [18]	Phage genome (or contigs)	Genus	RefSeq [83]	Accuracy = 59%
PHP [17]	Phage genome (or contigs)	Genus	VHM dataset [10], NCBI Genome [86]	Accuracy = 34% (VHM dataset), 35% (NCBI Genome)
RaFAH [19]	Phage genome (or contigs)	Genus	RefSeq [83]	F1 = 59%
HoPhage [22]	Phage genome (or contigs)	Genus	RefSeq [83] + VHDB [85]	Accuracy = 81.11%
HostG [81]	Phage genome (or contigs)	Genus	PHP dataset [17]	100% accuracy at softmax threshold of 94%
DeepHost [23]	Phage genome	Species	NCBI Genome [86] + EMBL [87] + PhagesDB [82] + Phage evolution database [88]	Accuracy = 90.78%
Multiclass Classification (Selected Proteins as Input)				
Boeckaerts *et al*. [15]	Phage receptor-binding proteins	Species	UniProtKB [89] + UniRef [40] + Millardlab dataset [90]	AUPR between 73.6% and 93.8% under different sequence similarity thresholds

* Datasets separated by a plus (+) indicate that selected entries from these datasets were aggregated to create a single test set.

^†^ PHISDetector [16] also provides an option to input only the phage genome.

## Conclusion

In this study, we capitalized on representation learning to automatically encode receptor-binding protein sequences into meaningful dense embeddings. To this end, we extensively tested different protein language models and built a random forest model for phage-host interaction prediction. Our experiments showed that the use of embeddings of receptor-binding proteins presents improvements over handcrafted genomic and protein sequence features, with the highest performance obtained using the transformer-based autoencoder model ProtT5. Moreover, these protein embeddings are able to capture complex combinations of biological features given only the raw sequences, without the need to supply additional alignment or structural information.

Our work makes the simplifying assumption that all the RBPs of a given phage are specific to one host. Albeit significantly less common than single-host phages, some phages are known to possess multiple RBPs, with the RBPs possibly adsorbing with different bacteria. For example, the polyvalent bacteriophage ΦK64–1 has eleven known RBPs targeting a wide spectrum of *Klebsiella* capsular types [91]. However, to the best of our knowledge, there are currently no existing datasets that map individual RBPs to their target hosts.

The receptor-binding proteins considered in our study are also limited to those of tailed phages belonging to the order *Caudovirales*, which constitute around 96% of all known phages [92]. It may also be interesting to explore a similar approach for the computational prediction of interaction between non-tailed phages and their hosts.

Further future directions include improving the interpretability of protein embeddings and incorporating other mechanisms related to phage-host interaction (e.g., restriction-modification and CRISPR-Cas systems), as well as host sequence information possibly encoded as dense embeddings.

## Glossary

- Autoencoding transformer—A type of transformer that is pretrained in a “bidirectional” fashion, i.e., it can read all the input tokens during pretraining
- Autoregressive transformer—A type of transformer that is pretrained in a “unidirectional” fashion, i.e., it attempts to predict the next token given only the previous tokens
- Extreme gradient boosting—A machine learning algorithm that iteratively combines weak “learners” (in this case, decision trees) to improve performance
- Hidden Markov model—A statistical model for modeling systems (including biological sequences) characterized by a sequence of observations and unobserved (hidden) states
- Protein language model—A deep learning model that adopts the architecture of models from natural language processing in order to convert protein sequences into dense vector representations (embeddings)
- Random forest—A machine learning algorithm that employs an ensemble of multiple decision trees to produce an output
- RBP—Receptor-binding protein. A specific type of protein found in tailed phages that is responsible for initiating the recognition and infection of bacterial hosts
- Transformer—A deep learning architecture characterized by the use of attention mechanisms to improve performance
- *t*-SNE—*t*-distributed stochastic neighbor embedding. A nonlinear method for projecting high-dimensional vectors onto a low-dimensional space by calculating a joint probability distribution that captures the similarity between data points
- UMAP—Uniform manifold approximation and projection. A nonlinear method for projecting high-dimensional vectors onto a low-dimensional space by assuming that the data points are evenly distributed on some topological space

## Supporting information

S1 File (PDF)
