# Supplementary material for: Protein embeddings improve phage-host interaction prediction
Source: PLoS One. 2023 Jul 24;18(7):e0289030. doi: 10.1371/journal.pone.0289030 (PMC10365317; doi:10.1371/journal.pone.0289030)
Supplement: S1 File — (PDF) [file pone.0289030.s001.pdf]

S1 Listing. Regular expression for the selection of annotated receptor-binding proteins. We modified the regular expression from Boeckaerts *et al.* [50] to better accommodate typographical errors and naming variations in the gene product annotations in GenBank.

```
tail?(.?|\s*)(?:spike?|fib(?:er|re))|recept(?:o|e)r(.?|\s*)(?:bind|recogn)
.*(?:protein)?|(?<!\w)RBP(?!a)
```

S1 Table. Number of training and test samples for all class labels. Note that *others* is not a class label in the training set; rather, it indicates that a given sample falls outside the class labels for which the model was trained.

Host	Training Set	Test Set	Total
Escherichia	3,021	1,295	4,316
Salmonella	1,474	632	2,106
Synechococcus	1,216	521	1,737
Pseudomonas	1,196	513	1,709
Vibrio	1,079	463	1,542
Klebsiella	926	397	1,323
Erwinia	667	286	953
Mycobacterium	578	248	826
Staphylococcus	568	244	812
Bacillus	475	204	679
Rheinheimera	409	176	585
Shigella	332	143	475
Enterobacter	330	142	472
Campylobacter	290	124	414
Lactococcus	233	100	333
Acinetobacter	230	98	328
Serratia	226	97	323
Aeromonas	223	95	318
Yersinia	218	93	311
Streptococcus	190	81	271
Rhizobium	174	74	248
Pectobacterium	167	71	238
Xanthomonas	136	58	194
Gordonia	123	53	176
Prochlorococcus	123	52	175
Dickeya	121	52	173
Enterococcus	114	49	163
Flavobacterium	113	48	161
Streptomyces	101	43	144
Ralstonia	99	42	141
Stenotrophomonas	97	41	138
Proteus	92	40	132
Burkholderia	90	39	129
Cronobacter	84	36	120
Rhodobacter	79	34	113
Citrobacter	78	34	112
Microbacterium	76	33	109
Pelagibacter	76	33	109
Bacteroides	72	31	103
Listeria	67	28	95
Arthrobacter	61	26	87
Caulobacter	60	25	85
Clostridium	55	24	79
Lactobacillus	54	23	77
Prevotella	43	18	61
Achromobacter	41	17	58

Continued on the next page

Host	Training Set	Test Set	Total
Pseudoalteromonas	39	17	56
Pantoea	38	16	54
Providencia	38	16	54
Helicobacter	36	16	52
Rhodococcus	32	13	45
Halorubrum	29	13	42
Agrobacterium	29	12	41
Shewanella	26	11	37
Leuconostoc	24	10	34
Kosakonia	24	10	34
Edwardsiella	22	10	32
Brevundimonas	22	10	32
Others	0	986	986
Total	16,636	8,116	24,752

S2 Table. Definition of true and false positive and negative outcomes. p_1 and p_2 are the highest and second-highest class probabilities for a given sample, and k is a given confidence threshold. A sample is classified under its (original) predicted class label if and only if $p_1 - p_2 \geq k$. If $p_1 - p_2 < k$, it is classified as *others*. X and Y denote two distinct class labels; *others* indicates that the sample falls outside the class labels for which the model was trained. *TP*, *FP*, *TN*, and *FN* stand for *true positive*, *false positive*, *true negative*, and *false negative*, respectively.

$p_1 - p_2$	True Label	Predicted Label (Original)	Predicted Label (In View of $p_1 - p_2$)	Evaluation Outcome
$\geq k$	X	X	X	TP for X
	Y	X	X	FN for Y<br>FP for X
	others	X	X	FN for *others*<br>FP for X
$< k$	X	X	*others*	FN for X<br>FP for *others*
	Y	X	*others*	FN for Y<br>FP for *others*<br>TN for X
	others	X	*others*	TP for *others*<br>TN for X

S3 Table. Model performance in terms of weighted precision. The header row refers to the confidence thresholds at which we evaluated model performance. These confidence thresholds range from $k = 60\%$ to 100% in steps of 10%. The highest weighted precision scores are given in bold and underlined.

	60%	70%	80%	90%	100%
Boeckaerts *et al.* [15]	85.55%	84.88%	84.05%	83.58%	73.09%
SeqVec	85.65%	84.56%	84.26%	<u>**83.83%**</u>	73.20%
ESM	85.31%	84.62%	83.66%	82.68%	76.51%
ESM-1b	84.71%	<u>**84.99%**</u>	<u>**84.40%**</u>	83.42%	76.89%
ProtBert	84.96%	84.55%	84.20%	83.69%	73.74%
ProtXLNet	<u>**85.66%**</u>	84.67%	84.06%	82.80%	77.07%
ProtAlbert	84.91%	84.61%	83.97%	83.03%	76.06%
ProtT5	85.43%	84.98%	84.32%	83.51%	<u>**77.23%**</u>

S4 Table. Model performance in terms of weighted recall. The header row refers to the confidence thresholds at which we evaluated model performance. These confidence thresholds range from $k = 60\%$ to 100% in steps of 10%. The highest weighted recall scores are given in bold and underlined.

	60%	70%	80%	90%	100%
Boeckaerts *et al.* [15]	55.48%	49.84%	44.50%	37.81%	24.84%
SeqVec	56.73%	51.22%	45.97%	38.87%	25.89%
ESM	58.33%	53.38%	47.92%	40.69%	<u>**27.34%**</u>
ESM-1b	58.49%	53.30%	47.86%	40.59%	27.24%
ProtBert	56.65%	51.60%	46.16%	39.48%	27.02%
ProtXLNet	56.32%	50.95%	45.50%	38.50%	26.56%
ProtAlbert	56.67%	51.21%	45.53%	38.90%	26.38%
ProtT5	<u>**59.15%**</u>	<u>**53.72%**</u>	<u>**48.57%**</u>	<u>**41.03%**</u>	27.16%

S5 Table. Model performance in terms of weighted specificity. The header row refers to the confidence thresholds at which we evaluated model performance. These confidence thresholds range from $k = 60\%$ to 100% in steps of 10%. The highest weighted specificity scores are given in bold and underlined.

	60%	70%	80%	90%	100%
Boeckaerts *et al.* [15]	93.89%	93.10%	92.36%	91.41%	89.61%
SeqVec	94.06%	93.29%	92.56%	91.56%	89.76%
ESM	94.33%	93.60%	92.83%	91.82%	<u>**89.96%**</u>
ESM-1b	94.35%	93.60%	92.83%	91.81%	89.94%
ProtBert	94.08%	93.36%	92.59%	91.64%	89.91%
ProtXLNet	94.04%	93.26%	92.50%	91.52%	89.85%
ProtAlbert	94.08%	93.30%	92.50%	91.57%	89.82%
ProtT5	<u>**94.44%**</u>	<u>**93.66%**</u>	<u>**92.93%**</u>	<u>**91.87%**</u>	89.93%

S6 Table. Per-class evaluation results of using ProtT5 embeddings at confidence threshold $k = 60\%$.

Host	Precision	Recall	F1	Specificity	Num. of Test Samples
Escherichia	90.94%	44.94%	60.16%	99.15%	1,295
Salmonella	94.44%	56.49%	70.69%	99.72%	632
Synechococcus	99.27%	77.93%	87.31%	99.96%	521
Pseudomonas	99.48%	74.07%	84.92%	99.97%	513
Vibrio	100.00%	53.56%	69.76%	100.00%	463
Klebsiella	95.94%	47.61%	63.64%	99.90%	397
Erwinia	96.73%	51.75%	67.43%	99.94%	286
Mycobacterium	98.66%	89.11%	93.64%	99.96%	248
Staphylococcus	100.00%	87.70%	93.45%	100.00%	244
Bacillus	96.38%	65.20%	77.78%	99.94%	204
Rheinheimera	100.00%	98.86%	99.43%	100.00%	176
Shigella	64.00%	11.19%	19.05%	99.89%	143
Enterobacter	76.19%	11.27%	19.63%	99.94%	142
Campylobacter	96.10%	59.68%	73.63%	99.96%	124
Lactococcus	100.00%	70.00%	82.35%	100.00%	100
Acinetobacter	100.00%	31.63%	48.06%	100.00%	98
Serratia	86.11%	31.96%	46.62%	99.94%	97
Aeromonas	100.00%	23.16%	37.61%	100.00%	95
Yersinia	77.27%	36.56%	49.64%	99.88%	93
Streptococcus	100.00%	58.02%	73.44%	100.00%	81
Rhizobium	100.00%	55.41%	71.30%	100.00%	74
Pectobacterium	97.06%	46.48%	62.86%	99.99%	71
Xanthomonas	94.74%	31.03%	46.75%	99.99%	58
Gordonia	100.00%	45.28%	62.34%	100.00%	53
Prochlorococcus	94.74%	34.62%	50.70%	99.99%	52
Dickeya	100.00%	48.08%	64.94%	100.00%	52
Enterococcus	100.00%	51.02%	67.57%	100.00%	49
Flavobacterium	100.00%	85.42%	92.13%	100.00%	48
Streptomyces	100.00%	46.51%	63.49%	100.00%	43
Ralstonia	100.00%	35.71%	52.63%	100.00%	42
Stenotrophomonas	100.00%	17.07%	29.17%	100.00%	41
Proteus	100.00%	20.00%	33.33%	100.00%	40
Burkholderia	100.00%	12.82%	22.73%	100.00%	39
Cronobacter	100.00%	5.56%	10.53%	100.00%	36
Rhodobacter	95.83%	67.65%	79.31%	99.99%	34
Citrobacter	100.00%	5.88%	11.11%	100.00%	34
Microbacterium	100.00%	51.52%	68.00%	100.00%	33
Pelagibacter	100.00%	24.24%	39.02%	100.00%	33
Bacteroides	100.00%	58.06%	73.47%	100.00%	31
Listeria	90.00%	64.29%	75.00%	99.98%	28
Arthrobacter	100.00%	50.00%	66.67%	100.00%	26
Caulobacter	100.00%	64.00%	78.05%	100.00%	25
Clostridium	66.67%	16.67%	26.67%	99.98%	24
Lactobacillus	50.00%	4.35%	8.00%	99.99%	23
Prevotella	100.00%	83.33%	90.91%	100.00%	18
Achromobacter	100.00%	11.76%	21.05%	100.00%	17
Pseudoalteromonas	100.00%	11.76%	21.05%	100.00%	17

Continued on the next page

Host	Precision	Recall	F1	Specificity	Num. of Test Samples
Pantoea	0.00%	0.00%	0.00%	100.00%	16
Providencia	66.67%	12.50%	21.05%	99.99%	16
Helicobacter	100.00%	87.50%	93.33%	100.00%	16
Rhodococcus	0.00%	0.00%	0.00%	100.00%	13
Halorubrum	30.77%	30.77%	30.77%	99.89%	13
Agrobacterium	100.00%	33.33%	50.00%	100.00%	12
Shewanella	0.00%	0.00%	0.00%	100.00%	11
Leuconostoc	100.00%	40.00%	57.14%	100.00%	10
Kosakonia	0.00%	0.00%	0.00%	100.00%	10
Edwardsiella	0.00%	0.00%	0.00%	100.00%	10
Brevundimonas	100.00%	20.00%	33.33%	100.00%	10
Others	23.25%	97.06%	37.51%	55.69%	986

S7 Table. Per-class evaluation results of using ProtT5 embeddings at confidence threshold $k = 70\%$.

Host	Precision	Recall	F1	Specificity	Num. of Test Samples
Escherichia	91.57%	37.76%	53.47%	99.34%	1,295
Salmonella	95.58%	47.94%	63.86%	99.81%	632
Synechococcus	99.49%	74.86%	85.43%	99.97%	521
Pseudomonas	99.71%	67.84%	80.74%	99.99%	513
Vibrio	100.00%	44.92%	62.00%	100.00%	463
Klebsiella	95.43%	42.07%	58.39%	99.90%	397
Erwinia	97.58%	42.31%	59.02%	99.96%	286
Mycobacterium	98.64%	87.90%	92.96%	99.96%	248
Staphylococcus	100.00%	84.43%	91.56%	100.00%	244
Bacillus	97.54%	58.33%	73.01%	99.96%	204
Rheinheimera	100.00%	97.73%	98.85%	100.00%	176
Shigella	63.64%	9.79%	16.97%	99.90%	143
Enterobacter	70.00%	4.93%	9.21%	99.96%	142
Campylobacter	96.88%	50.00%	65.96%	99.97%	124
Lactococcus	100.00%	63.00%	77.30%	100.00%	100
Acinetobacter	100.00%	21.43%	35.29%	100.00%	98
Serratia	81.48%	22.68%	35.48%	99.94%	97
Aeromonas	100.00%	15.79%	27.27%	100.00%	95
Yersinia	96.55%	30.11%	45.90%	99.99%	93
Streptococcus	100.00%	50.62%	67.21%	100.00%	81
Rhizobium	100.00%	41.89%	59.05%	100.00%	74
Pectobacterium	96.77%	42.25%	58.82%	99.99%	71
Xanthomonas	93.33%	24.14%	38.36%	99.99%	58
Gordonia	100.00%	37.74%	54.79%	100.00%	53
Prochlorococcus	93.33%	26.92%	41.79%	99.99%	52
Dickeya	100.00%	44.23%	61.33%	100.00%	52
Enterococcus	100.00%	46.94%	63.89%	100.00%	49
Flavobacterium	100.00%	85.42%	92.13%	100.00%	48
Streptomyces	100.00%	44.19%	61.29%	100.00%	43
Ralstonia	100.00%	26.19%	41.51%	100.00%	42
Stenotrophomonas	100.00%	12.20%	21.74%	100.00%	41
Proteus	100.00%	15.00%	26.09%	100.00%	40
Burkholderia	100.00%	10.26%	18.60%	100.00%	39

Continued on the next page

Host	Precision	Recall	F1	Specificity	Num. of Test Samples
Cronobacter	100.00%	2.78%	5.41%	100.00%	36
Rhodobacter	100.00%	64.71%	78.57%	100.00%	34
Citrobacter	0.00%	0.00%	0.00%	100.00%	34
Microbacterium	100.00%	42.42%	59.57%	100.00%	33
Pelagibacter	100.00%	9.09%	16.67%	100.00%	33
Bacteroides	100.00%	48.39%	65.22%	100.00%	31
Listeria	93.33%	50.00%	65.12%	99.99%	28
Arthrobacter	100.00%	46.15%	63.16%	100.00%	26
Caulobacter	100.00%	64.00%	78.05%	100.00%	25
Clostridium	66.67%	16.67%	26.67%	99.98%	24
Lactobacillus	0.00%	0.00%	0.00%	100.00%	23
Prevotella	100.00%	83.33%	90.91%	100.00%	18
Achromobacter	100.00%	5.88%	11.11%	100.00%	17
Pseudoalteromonas	100.00%	5.88%	11.11%	100.00%	17
Pantoea	0.00%	0.00%	0.00%	100.00%	16
Providencia	100.00%	6.25%	11.76%	100.00%	16
Helicobacter	100.00%	75.00%	85.71%	100.00%	16
Rhodococcus	0.00%	0.00%	0.00%	100.00%	13
Halorubrum	30.77%	30.77%	30.77%	99.89%	13
Agrobacterium	100.00%	25.00%	40.00%	100.00%	12
Shewanella	0.00%	0.00%	0.00%	100.00%	11
Leuconostoc	100.00%	10.00%	18.18%	100.00%	10
Kosakonia	0.00%	0.00%	0.00%	100.00%	10
Edwardsiella	0.00%	0.00%	0.00%	100.00%	10
Brevundimonas	100.00%	10.00%	18.18%	100.00%	10
Others	20.94%	97.87%	34.50%	48.91%	986

S8 Table. Per-class evaluation results of using ProtT5 embeddings at confidence threshold $k = 80\%$.

Host	Precision	Recall	F1	Specificity	Num. of Test Samples
Escherichia	93.37%	29.34%	44.65%	99.60%	1,295
Salmonella	95.76%	42.88%	59.23%	99.84%	632
Synechococcus	99.47%	72.55%	83.91%	99.97%	521
Pseudomonas	99.69%	63.16%	77.33%	99.99%	513
Vibrio	100.00%	39.96%	57.10%	100.00%	463
Klebsiella	97.54%	29.97%	45.86%	99.96%	397
Erwinia	98.88%	30.77%	46.93%	99.99%	286
Mycobacterium	98.61%	85.89%	91.81%	99.96%	248
Staphylococcus	100.00%	80.74%	89.34%	100.00%	244
Bacillus	98.02%	48.53%	64.92%	99.97%	204
Rheinheimera	100.00%	96.59%	98.27%	100.00%	176
Shigella	57.89%	7.69%	13.58%	99.90%	143
Enterobacter	57.14%	2.82%	5.37%	99.96%	142
Campylobacter	100.00%	37.10%	54.12%	100.00%	124
Lactococcus	100.00%	61.00%	75.78%	100.00%	100
Acinetobacter	100.00%	16.33%	28.07%	100.00%	98
Serratia	85.71%	18.56%	30.51%	99.96%	97
Aeromonas	100.00%	11.58%	20.75%	100.00%	95
Yersinia	96.15%	26.88%	42.02%	99.99%	93

Continued on the next page

Host	Precision	Recall	F1	Specificity	Num. of Test Samples
Streptococcus	100.00%	43.21%	60.34%	100.00%	81
Rhizobium	100.00%	31.08%	47.42%	100.00%	74
Pectobacterium	100.00%	36.62%	53.61%	100.00%	71
Xanthomonas	92.31%	20.69%	33.80%	99.99%	58
Gordonia	100.00%	24.53%	39.39%	100.00%	53
Prochlorococcus	100.00%	25.00%	40.00%	100.00%	52
Dickeya	100.00%	44.23%	61.33%	100.00%	52
Enterococcus	100.00%	44.90%	61.97%	100.00%	49
Flavobacterium	100.00%	83.33%	90.91%	100.00%	48
Streptomyces	100.00%	37.21%	54.24%	100.00%	43
Ralstonia	100.00%	23.81%	38.46%	100.00%	42
Stenotrophomonas	100.00%	7.32%	13.64%	100.00%	41
Proteus	100.00%	15.00%	26.09%	100.00%	40
Burkholderia	100.00%	7.69%	14.29%	100.00%	39
Cronobacter	100.00%	2.78%	5.41%	100.00%	36
Rhodobacter	100.00%	58.82%	74.07%	100.00%	34
Citrobacter	0.00%	0.00%	0.00%	100.00%	34
Microbacterium	100.00%	33.33%	50.00%	100.00%	33
Pelagibacter	100.00%	3.03%	5.88%	100.00%	33
Bacteroides	100.00%	29.03%	45.00%	100.00%	31
Listeria	100.00%	39.29%	56.41%	100.00%	28
Arthrobacter	100.00%	26.92%	42.42%	100.00%	26
Caulobacter	100.00%	64.00%	78.05%	100.00%	25
Clostridium	66.67%	8.33%	14.81%	99.99%	24
Lactobacillus	0.00%	0.00%	0.00%	100.00%	23
Prevotella	100.00%	83.33%	90.91%	100.00%	18
Achromobacter	0.00%	0.00%	0.00%	100.00%	17
Pseudoalteromonas	0.00%	0.00%	0.00%	100.00%	17
Pantoea	0.00%	0.00%	0.00%	100.00%	16
Providencia	0.00%	0.00%	0.00%	100.00%	16
Helicobacter	100.00%	68.75%	81.48%	100.00%	16
Rhodococcus	0.00%	0.00%	0.00%	100.00%	13
Halorubrum	25.00%	23.08%	24.00%	99.89%	13
Agrobacterium	100.00%	25.00%	40.00%	100.00%	12
Shewanella	0.00%	0.00%	0.00%	100.00%	11
Leuconostoc	0.00%	0.00%	0.00%	100.00%	10
Kosakonia	0.00%	0.00%	0.00%	100.00%	10
Edwardsiella	0.00%	0.00%	0.00%	100.00%	10
Brevundimonas	100.00%	10.00%	18.18%	100.00%	10
Others	19.14%	98.38%	32.05%	42.54%	986

S9 Table. Per-class evaluation results of using ProtT5 embeddings at confidence threshold $k = 90\%$.

Host	Precision	Recall	F1	Specificity	Num. of Test Samples
Escherichia	95.33%	18.92%	31.57%	99.82%	1,295
Salmonella	96.40%	33.86%	50.12%	99.89%	632
Synechococcus	99.44%	67.95%	80.73%	99.97%	521
Pseudomonas	100.00%	52.24%	68.63%	100.00%	513
Vibrio	100.00%	31.32%	47.70%	100.00%	463
Klebsiella	98.77%	20.15%	33.47%	99.99%	397
Erwinia	100.00%	23.43%	37.96%	100.00%	286
Mycobacterium	98.58%	84.27%	90.87%	99.96%	248
Staphylococcus	100.00%	70.90%	82.97%	100.00%	244
Bacillus	100.00%	31.37%	47.76%	100.00%	204
Rheinheimera	100.00%	96.59%	98.27%	100.00%	176
Shigella	37.50%	2.10%	3.97%	99.94%	143
Enterobacter	60.00%	2.11%	4.08%	99.97%	142
Campylobacter	100.00%	17.74%	30.14%	100.00%	124
Lactococcus	100.00%	57.00%	72.61%	100.00%	100
Acinetobacter	100.00%	8.16%	15.09%	100.00%	98
Serratia	88.89%	8.25%	15.09%	99.99%	97
Aeromonas	100.00%	3.16%	6.12%	100.00%	95
Yersinia	95.24%	21.51%	35.09%	99.99%	93
Streptococcus	100.00%	32.10%	48.60%	100.00%	81
Rhizobium	100.00%	21.62%	35.56%	100.00%	74
Pectobacterium	100.00%	30.99%	47.31%	100.00%	71
Xanthomonas	80.00%	6.90%	12.70%	99.99%	58
Gordonia	100.00%	15.09%	26.23%	100.00%	53
Prochlorococcus	100.00%	23.08%	37.50%	100.00%	52
Dickeya	100.00%	36.54%	53.52%	100.00%	52
Enterococcus	100.00%	44.90%	61.97%	100.00%	49
Flavobacterium	100.00%	70.83%	82.93%	100.00%	48
Streptomyces	100.00%	20.93%	34.62%	100.00%	43
Ralstonia	100.00%	7.14%	13.33%	100.00%	42
Stenotrophomonas	100.00%	4.88%	9.30%	100.00%	41
Proteus	100.00%	10.00%	18.18%	100.00%	40
Burkholderia	100.00%	5.13%	9.76%	100.00%	39
Cronobacter	0.00%	0.00%	0.00%	100.00%	36
Rhodobacter	100.00%	41.18%	58.33%	100.00%	34
Citrobacter	0.00%	0.00%	0.00%	100.00%	34
Microbacterium	100.00%	15.15%	26.32%	100.00%	33
Pelagibacter	100.00%	3.03%	5.88%	100.00%	33
Bacteroides	100.00%	3.23%	6.25%	100.00%	31
Listeria	100.00%	25.00%	40.00%	100.00%	28
Arthrobacter	100.00%	11.54%	20.69%	100.00%	26
Caulobacter	100.00%	32.00%	48.48%	100.00%	25
Clostridium	66.67%	8.33%	14.81%	99.99%	24
Lactobacillus	0.00%	0.00%	0.00%	100.00%	23
Prevotella	100.00%	66.67%	80.00%	100.00%	18
Achromobacter	0.00%	0.00%	0.00%	100.00%	17
Pseudoalteromonas	0.00%	0.00%	0.00%	100.00%	17

Continued on the next page

Host	Precision	Recall	F1	Specificity	Num. of Test Samples
Pantoea	0.00%	0.00%	0.00%	100.00%	16
Providencia	0.00%	0.00%	0.00%	100.00%	16
Helicobacter	100.00%	18.75%	31.58%	100.00%	16
Rhodococcus	0.00%	0.00%	0.00%	100.00%	13
Halorubrum	20.00%	7.69%	11.11%	99.95%	13
Agrobacterium	0.00%	0.00%	0.00%	100.00%	12
Shewanella	0.00%	0.00%	0.00%	100.00%	11
Leuconostoc	0.00%	0.00%	0.00%	100.00%	10
Kosakonia	0.00%	0.00%	0.00%	100.00%	10
Edwardsiella	0.00%	0.00%	0.00%	100.00%	10
Brevundimonas	0.00%	0.00%	0.00%	100.00%	10
Others	17.07%	99.09%	29.13%	33.45%	986

S10 Table. Per-class evaluation results of using ProtT5 embeddings at confidence threshold $k = 100\%$.

Host	Precision	Recall	F1	Specificity	Num. of Test Samples
Escherichia	94.57%	6.72%	12.55%	99.93%	1,295
Salmonella	100.00%	12.82%	22.72%	100.00%	632
Synechococcus	99.63%	51.25%	67.68%	99.99%	521
Pseudomonas	100.00%	30.02%	46.18%	100.00%	513
Vibrio	100.00%	14.90%	25.94%	100.00%	463
Klebsiella	100.00%	4.03%	7.75%	100.00%	397
Erwinia	100.00%	3.85%	7.41%	100.00%	286
Mycobacterium	98.20%	66.13%	79.04%	99.96%	248
Staphylococcus	100.00%	29.51%	45.57%	100.00%	244
Bacillus	100.00%	4.90%	9.35%	100.00%	204
Rheinheimera	100.00%	93.18%	96.47%	100.00%	176
Shigella	100.00%	0.70%	1.39%	100.00%	143
Enterobacter	0.00%	0.00%	0.00%	100.00%	142
Campylobacter	100.00%	4.84%	9.23%	100.00%	124
Lactococcus	100.00%	23.00%	37.40%	100.00%	100
Acinetobacter	100.00%	1.02%	2.02%	100.00%	98
Serratia	100.00%	2.06%	4.04%	100.00%	97
Aeromonas	0.00%	0.00%	0.00%	100.00%	95
Yersinia	100.00%	11.83%	21.15%	100.00%	93
Streptococcus	100.00%	2.47%	4.82%	100.00%	81
Rhizobium	0.00%	0.00%	0.00%	100.00%	74
Pectobacterium	100.00%	11.27%	20.25%	100.00%	71
Xanthomonas	50.00%	1.72%	3.33%	99.99%	58
Gordonia	0.00%	0.00%	0.00%	100.00%	53
Prochlorococcus	100.00%	21.15%	34.92%	100.00%	52
Dickeya	100.00%	28.85%	44.78%	100.00%	52
Enterococcus	100.00%	6.12%	11.54%	100.00%	49
Flavobacterium	100.00%	62.50%	76.92%	100.00%	48
Streptomyces	100.00%	6.98%	13.04%	100.00%	43
Ralstonia	0.00%	0.00%	0.00%	100.00%	42
Stenotrophomonas	0.00%	0.00%	0.00%	100.00%	41
Proteus	100.00%	5.00%	9.52%	100.00%	40
Burkholderia	0.00%	0.00%	0.00%	100.00%	39

Continued on the next page

Host	Precision	Recall	F1	Specificity	Num. of Test Samples
Cronobacter	0.00%	0.00%	0.00%	100.00%	36
Rhodobacter	100.00%	14.71%	25.64%	100.00%	34
Citrobacter	0.00%	0.00%	0.00%	100.00%	34
Microbacterium	0.00%	0.00%	0.00%	100.00%	33
Pelagibacter	0.00%	0.00%	0.00%	100.00%	33
Bacteroides	0.00%	0.00%	0.00%	100.00%	31
Listeria	100.00%	3.57%	6.90%	100.00%	28
Arthrobacter	0.00%	0.00%	0.00%	100.00%	26
Caulobacter	100.00%	4.00%	7.69%	100.00%	25
Clostridium	0.00%	0.00%	0.00%	100.00%	24
Lactobacillus	0.00%	0.00%	0.00%	100.00%	23
Prevotella	100.00%	5.56%	10.53%	100.00%	18
Achromobacter	0.00%	0.00%	0.00%	100.00%	17
Pseudoalteromonas	0.00%	0.00%	0.00%	100.00%	17
Pantoea	0.00%	0.00%	0.00%	100.00%	16
Providencia	0.00%	0.00%	0.00%	100.00%	16
Helicobacter	0.00%	0.00%	0.00%	100.00%	16
Rhodococcus	0.00%	0.00%	0.00%	100.00%	13
Halorubrum	0.00%	0.00%	0.00%	100.00%	13
Agrobacterium	0.00%	0.00%	0.00%	100.00%	12
Shewanella	0.00%	0.00%	0.00%	100.00%	11
Leuconostoc	0.00%	0.00%	0.00%	100.00%	10
Kosakonia	0.00%	0.00%	0.00%	100.00%	10
Edwardsiella	0.00%	0.00%	0.00%	100.00%	10
Brevundimonas	0.00%	0.00%	0.00%	100.00%	10
Others	14.26%	99.59%	24.96%	17.22%	986

S11 Table. Weighted precision scores after integrating handcrafted sequence properties to the vector representations of the receptor-binding proteins. The selected sequence properties are those with the highest Gini importance after training the phage-host interaction prediction tool by Boeckaerts *et al.* [15] on our dataset. The header row refers to the confidence thresholds at which we evaluated model performance.

	60%	70%	80%	90%	100%
ProtT5	85.43%	84.98%	84.32%	83.51%	77.23%
ProtT5 + A Nucleotide Frequency	85.86%	85.05%	84.48%	83.47%	76.52%
ProtT5 + GC Content	85.89%	84.93%	84.22%	84.10%	75.47%
ProtT5 + C Nucleotide Frequency	85.44%	84.80%	84.43%	83.79%	77.00%
ProtT5 + TTA Codon Frequency	85.46%	84.81%	84.34%	83.60%	77.03%
ProtT5 + TTA Codon Usage Bias	85.69%	84.78%	84.89%	84.01%	75.16%

S12 Table. Weighted precision scores after integrating handcrafted protein sequence properties to the vector representations of the receptor-binding proteins. The selected protein sequence properties are those with the highest Gini importance after training the phage-host interaction prediction tool by Boeckaerts *et al.* [15] on our dataset. The header row refers to the confidence thresholds at which we evaluated model performance.

	60%	70%	80%	90%	100%
ProtT5	85.43%	84.98%	84.32%	83.51%	77.23%
ProtT5 + K (Lysine) Frequency	85.54%	84.87%	84.24%	83.98%	78.06%
ProtT5 + Isoelectric Point (pI)	85.28%	85.34%	84.40%	83.71%	77.09%
ProtT5 + Fourth Protein Z-Scale*	85.37%	84.77%	84.32%	83.79%	77.44%
ProtT5 + % of Exposed SA†	84.95%	84.96%	84.34%	83.47%	76.82%
ProtT5 + Molecular Weight	85.17%	85.06%	84.33%	83.26%	75.83%

* The fourth protein Z-scale (Z4) [60] is related to the heat of formation, hardness, electronegativity, and electrophilicity.
† *% of Exposed SA* refers to the percentage of residues with exposed solvent accessibility.

S13 Table. Weighted precision scores after integrating the top *n* handcrafted sequence properties to the vector representations of the receptor-binding proteins. The selected sequence properties are those with the highest Gini importance after training the phage-host interaction prediction tool by Boeckaerts *et al.* [15] on our dataset. These properties (in order of decreasing importance) are the A nucleotide frequency, GC content, C nucleotide frequency, TTA codon frequency, and TTA codon usage bias. The header row refers to the confidence thresholds at which we evaluated model performance.

	60%	70%	80%	90%	100%
ProtT5	85.43%	84.98%	84.32%	83.51%	77.23%
ProtT5 + Top 1	85.86%	85.05%	84.48%	83.47%	76.52%
ProtT5 + Top 2	85.80%	85.01%	84.59%	84.14%	76.98%
ProtT5 + Top 3	85.99%	85.18%	84.44%	84.31%	78.18%
ProtT5 + Top 4	85.46%	85.44%	84.41%	84.26%	74.61%
ProtT5 + Top 5	85.31%	85.58%	84.65%	84.11%	77.88%

S14 Table. Weighted precision scores after integrating the top n handcrafted protein sequence properties to the vector representations of the receptor-binding proteins. The selected protein sequence properties are those with the highest Gini importance after training the phage-host interaction prediction tool by Boeckaerts *et al.* [15] on our dataset. These properties (in order of decreasing importance) are the K (lysine) frequency, isoelectric point, fourth protein Z-scale [60] (which is related to the heat of formation, hardness, electronegativity, and electrophilicity), percentage of residues with exposed solvent accessibility, and molecular weight. The header row refers to the confidence thresholds at which we evaluated model performance.

	60%	70%	80%	90%	100%
ProtT5	85.43%	84.98%	84.32%	83.51%	77.23%
ProtT5 + Protein Top 1	85.54%	84.87%	84.24%	83.98%	78.06%
ProtT5 + Protein Top 2	85.53%	84.88%	84.29%	83.24%	75.05%
ProtT5 + Protein Top 3	85.19%	85.32%	84.12%	83.90%	78.62%
ProtT5 + Protein Top 4	85.18%	84.98%	83.99%	83.86%	77.62%
ProtT5 + Protein Top 5	85.42%	85.29%	84.51%	83.83%	75.81%

S15 Table. Weighted recall scores after integrating handcrafted sequence properties to the vector representations of the receptor-binding proteins. The selected sequence properties are those with the highest Gini importance after training the phage-host interaction prediction tool by Boeckaerts *et al.* [15] on our dataset. The header row refers to the confidence thresholds at which we evaluated model performance.

	60%	70%	80%	90%	100%
ProtT5	59.15%	53.72%	48.57%	41.03%	27.16%
ProtT5 + A Nucleotide Frequency	59.71%	54.51%	49.14%	41.29%	27.16%
ProtT5 + GC Content	60.20%	54.77%	49.15%	41.74%	27.70%
ProtT5 + C Nucleotide Frequency	59.91%	54.62%	48.98%	41.38%	27.24%
ProtT5 + TTA Codon Frequency	59.51%	53.77%	48.39%	41.01%	27.23%
ProtT5 + TTA Codon Usage Bias	59.41%	53.78%	48.32%	40.99%	27.07%

S16 Table. Weighted recall scores after integrating handcrafted protein sequence properties to the vector representations of the receptor-binding proteins. The selected protein sequence properties are those with the highest Gini importance after training the phage-host interaction prediction tool by Boeckaerts *et al.* [15] on our dataset. The header row refers to the confidence thresholds at which we evaluated model performance.

	60%	70%	80%	90%	100%
ProtT5	59.15%	53.72%	48.57%	41.03%	27.16%
ProtT5 + K (Lysine) Frequency	59.22%	53.67%	48.63%	41.13%	27.23%
ProtT5 + Isoelectric Point (pI)	59.13%	53.55%	48.44%	40.93%	27.51%
ProtT5 + Fourth Protein Z-Scale*	59.09%	53.63%	48.18%	41.01%	27.23%
ProtT5 + % of Exposed SA†	59.01%	53.82%	48.36%	40.88%	27.58%
ProtT5 + Molecular Weight	59.22%	53.87%	48.46%	40.86%	27.34%

* The fourth protein Z-scale (Z4) [60] is related to the heat of formation, hardness, electronegativity, and electrophilicity.
† *% of Exposed SA* refers to the percentage of residues with exposed solvent accessibility.

S17 Table. Weighted recall scores after integrating the top n handcrafted sequence properties to the vector representations of the receptor-binding proteins. The selected sequence properties are those with the highest Gini importance after training the phage-host interaction prediction tool by Boeckaerts *et al.* [15] on our dataset. These properties (in order of decreasing importance) are the A nucleotide frequency, GC content, C nucleotide frequency, TTA codon frequency, and TTA codon usage bias. The header row refers to the confidence thresholds at which we evaluated model performance.

	60%	70%	80%	90%	100%
ProtT5	59.15%	53.72%	48.57%	41.03%	27.16%
ProtT5 + Top 1	59.71%	54.51%	49.14%	41.29%	27.16%
ProtT5 + Top 2	60.55%	54.95%	49.22%	41.51%	27.37%
ProtT5 + Top 3	60.45%	55.01%	49.37%	41.93%	27.39%
ProtT5 + Top 4	60.31%	55.04%	49.25%	41.67%	26.81%
ProtT5 + Top 5	60.33%	54.87%	49.33%	41.94%	27.13%

S18 Table. Weighted recall scores after integrating the top n handcrafted protein sequence properties to the vector representations of the receptor-binding proteins. The selected protein sequence properties are those with the highest Gini importance after training the phage-host interaction prediction tool by Boeckaerts *et al.* [15] on our dataset. These properties (in order of decreasing importance) are the K (lysine) frequency, isoelectric point, fourth protein Z-scale [60] (which is related to the heat of formation, hardness, electronegativity, and electrophilicity), percentage of residues with exposed solvent accessibility, and molecular weight. The header row refers to the confidence thresholds at which we evaluated model performance.

	60%	70%	80%	90%	100%
ProtT5	59.15%	53.72%	48.57%	41.03%	27.16%
ProtT5 + Protein Top 1	59.22%	53.67%	48.63%	41.13%	27.23%
ProtT5 + Protein Top 2	59.23%	53.82%	48.47%	40.91%	27.37%
ProtT5 + Protein Top 3	59.15%	53.63%	48.10%	41.03%	27.44%
ProtT5 + Protein Top 4	59.19%	53.84%	48.34%	41.26%	27.53%
ProtT5 + Protein Top 5	58.99%	54.00%	48.51%	40.89%	27.11%

S19 Table. Weighted specificity scores after integrating handcrafted sequence properties to the vector representations of the receptor-binding proteins. The selected sequence properties are those with the highest Gini importance after training the phage-host interaction prediction tool by Boeckaerts *et al.* [15] on our dataset. The header row refers to the confidence thresholds at which we evaluated model performance.

	60%	70%	80%	90%	100%
ProtT5	94.44%	93.66%	92.93%	91.87%	89.93%
ProtT5 + A Nucleotide Frequency	94.51%	93.76%	93.00%	91.90%	89.93%
ProtT5 + GC Content	94.57%	93.80%	93.01%	91.97%	90.01%
ProtT5 + C Nucleotide Frequency	94.53%	93.78%	92.99%	91.92%	89.95%
ProtT5 + TTA Codon Frequency	94.48%	93.67%	92.90%	91.87%	89.94%
ProtT5 + TTA Codon Usage Bias	94.47%	93.67%	92.89%	91.86%	89.92%

S20 Table. Weighted specificity scores after integrating handcrafted protein sequence properties to the vector representations of the receptor-binding proteins. The selected protein sequence properties are those with the highest Gini importance after training the phage-host interaction prediction tool by Boeckaerts *et al.* [15] on our dataset. The header row refers to the confidence thresholds at which we evaluated model performance.

	60%	70%	80%	90%	100%
ProtT5	94.44%	93.66%	92.93%	91.87%	89.93%
ProtT5 + K (Lysine) Frequency	94.44%	93.65%	92.94%	91.89%	89.94%
ProtT5 + Isoelectric Point (pI)	94.43%	93.63%	92.91%	91.86%	89.98%
ProtT5 + Fourth Protein Z-Scale*	94.43%	93.64%	92.88%	91.87%	89.94%
ProtT5 + % of Exposed SA†	94.42%	93.67%	92.90%	91.85%	89.99%
ProtT5 + Molecular Weight	94.45%	93.68%	92.91%	91.85%	89.96%

* The fourth protein Z-scale (Z4) [60] is related to the heat of formation, hardness, electronegativity, and electrophilicity.

† *% of Exposed SA* refers to the percentage of residues with exposed solvent accessibility.

S21 Table. Weighted specificity scores after integrating the top n handcrafted sequence properties to the vector representations of the receptor-binding proteins. The selected sequence properties are those with the highest Gini importance after training the phage-host interaction prediction tool by Boeckaerts *et al.* [15] on our dataset. These properties (in order of decreasing importance) are the A nucleotide frequency, GC content, C nucleotide frequency, TTA codon frequency, and TTA codon usage bias. The header row refers to the confidence thresholds at which we evaluated model performance.

	60%	70%	80%	90%	100%
ProtT5	94.44%	93.66%	92.93%	91.87%	89.93%
ProtT5 + Top 1	94.51%	93.76%	93.00%	91.90%	89.93%
ProtT5 + Top 2	94.61%	93.83%	93.01%	91.94%	89.96%
ProtT5 + Top 3	94.60%	93.84%	93.03%	91.99%	89.96%
ProtT5 + Top 4	94.59%	93.83%	93.02%	91.96%	89.88%
ProtT5 + Top 5	94.59%	93.81%	93.03%	91.99%	89.93%

S22 Table. Weighted specificity scores after integrating the top n handcrafted protein sequence properties to the vector representations of the receptor-binding proteins. The selected protein sequence properties are those with the highest Gini importance after training the phage-host interaction prediction tool by Boeckaerts *et al.* [15] on our dataset. These properties (in order of decreasing importance) are the K (lysine) frequency, isoelectric point, fourth protein Z-scale [60] (which is related to the heat of formation, hardness, electronegativity, and electrophilicity), percentage of residues with exposed solvent accessibility, and molecular weight. The header row refers to the confidence thresholds at which we evaluated model performance.

	60%	70%	80%	90%	100%
ProtT5	94.44%	93.66%	92.93%	91.87%	89.93%
ProtT5 + Protein Top 1	94.44%	93.65%	92.94%	91.89%	89.94%
ProtT5 + Protein Top 2	94.45%	93.67%	92.91%	91.86%	89.96%
ProtT5 + Protein Top 3	94.44%	93.65%	92.86%	91.87%	89.97%
ProtT5 + Protein Top 4	94.45%	93.67%	92.90%	91.91%	89.98%
ProtT5 + Protein Top 5	94.41%	93.69%	92.92%	91.86%	89.92%

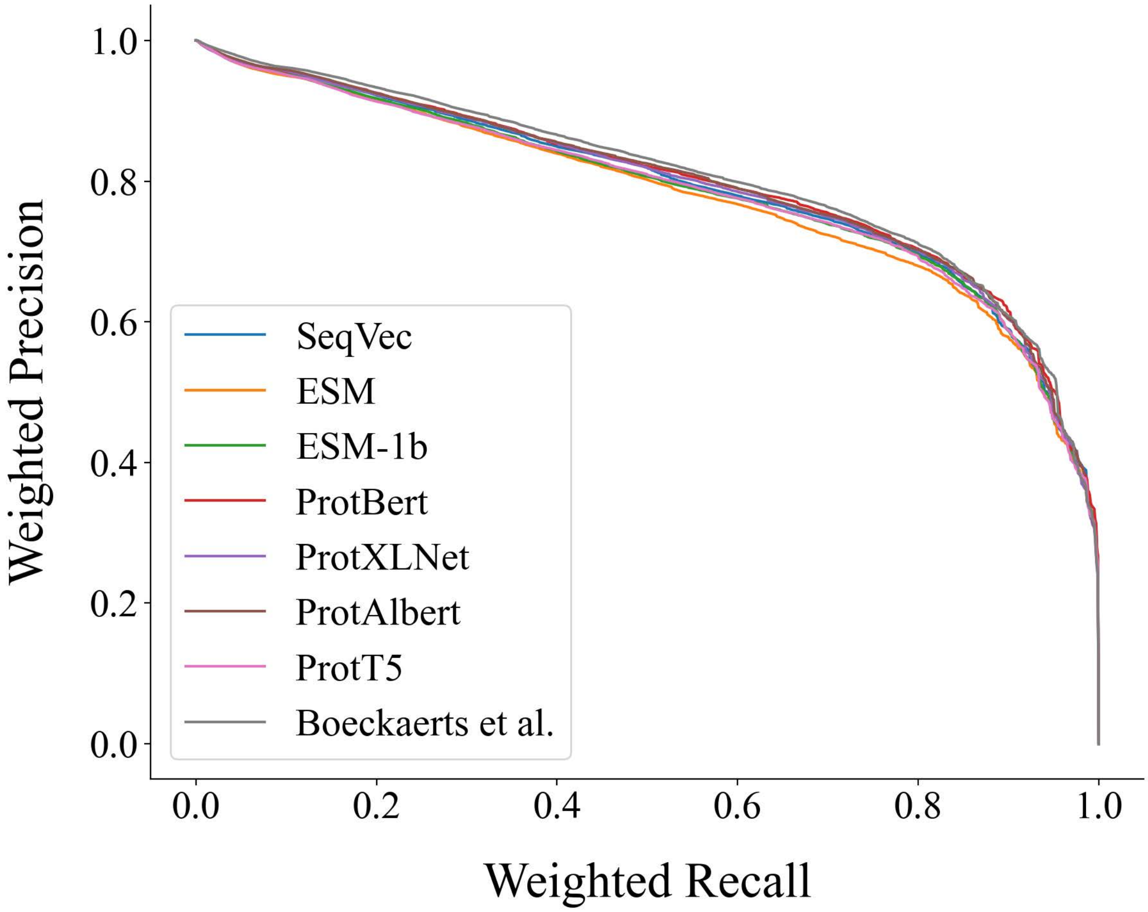

S1 Figure. Weighted precision-recall curves showing the model performance at $\boldsymbol{k = 0}$. At $k = 0$, none of the samples is labeled as *others*, i.e., the predicted class is the class with the highest class probability. The curves were generated following a one-versus-rest binarization of the predicted classes; the definitions of true and false positive and negative outcomes thus follow the standard definitions in binary classification. The precision and recall scores were then averaged, with the weights corresponding to the class sizes.

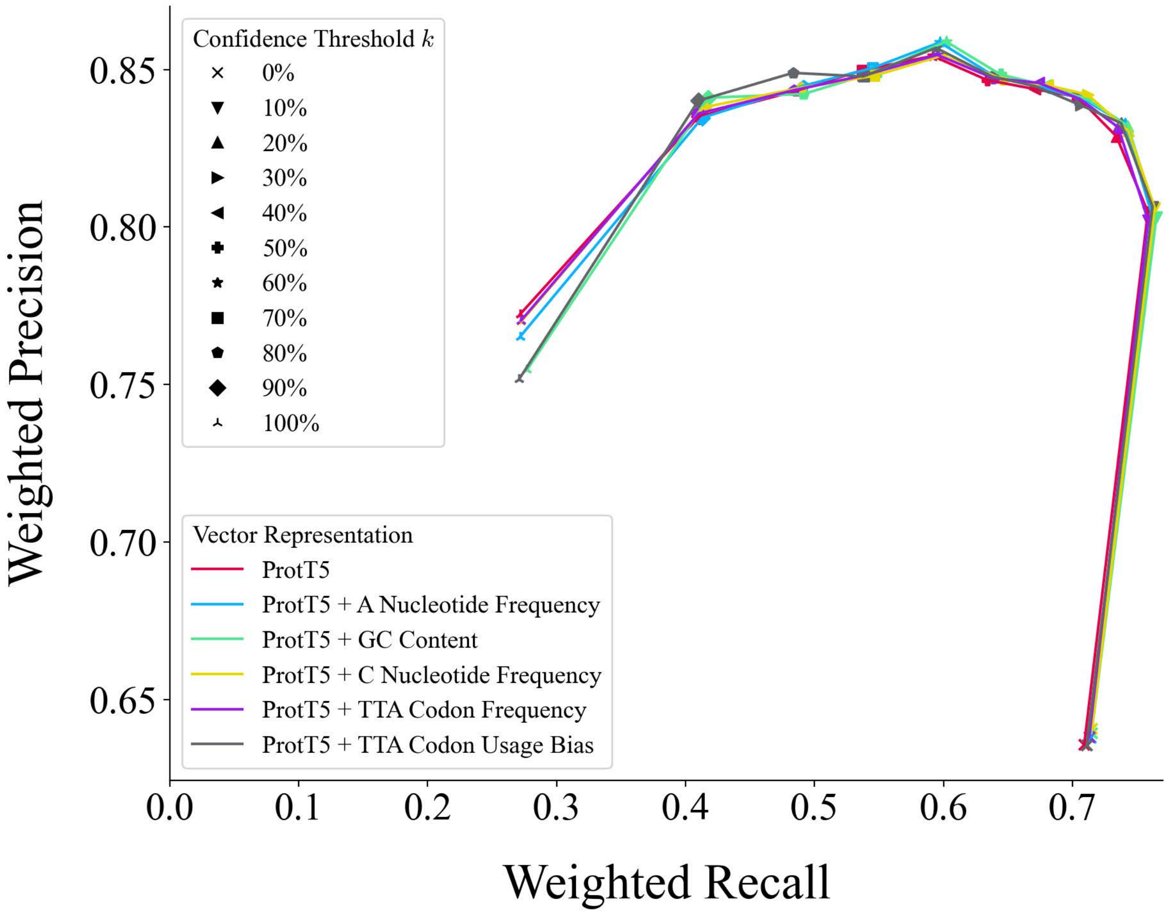

S2 Figure. Weighted precision-recall curves after integrating handcrafted sequence properties to the vector representations of the RBPs. The selected sequence properties are those with the highest Gini importance after training the phage-host interaction prediction tool by Boeckaerts *et al.* [15] on our dataset. The curves plot the weighted precision against the weighted recall at different confidence thresholds ranging from $k = 0\%$ to 100% in steps of 10%.

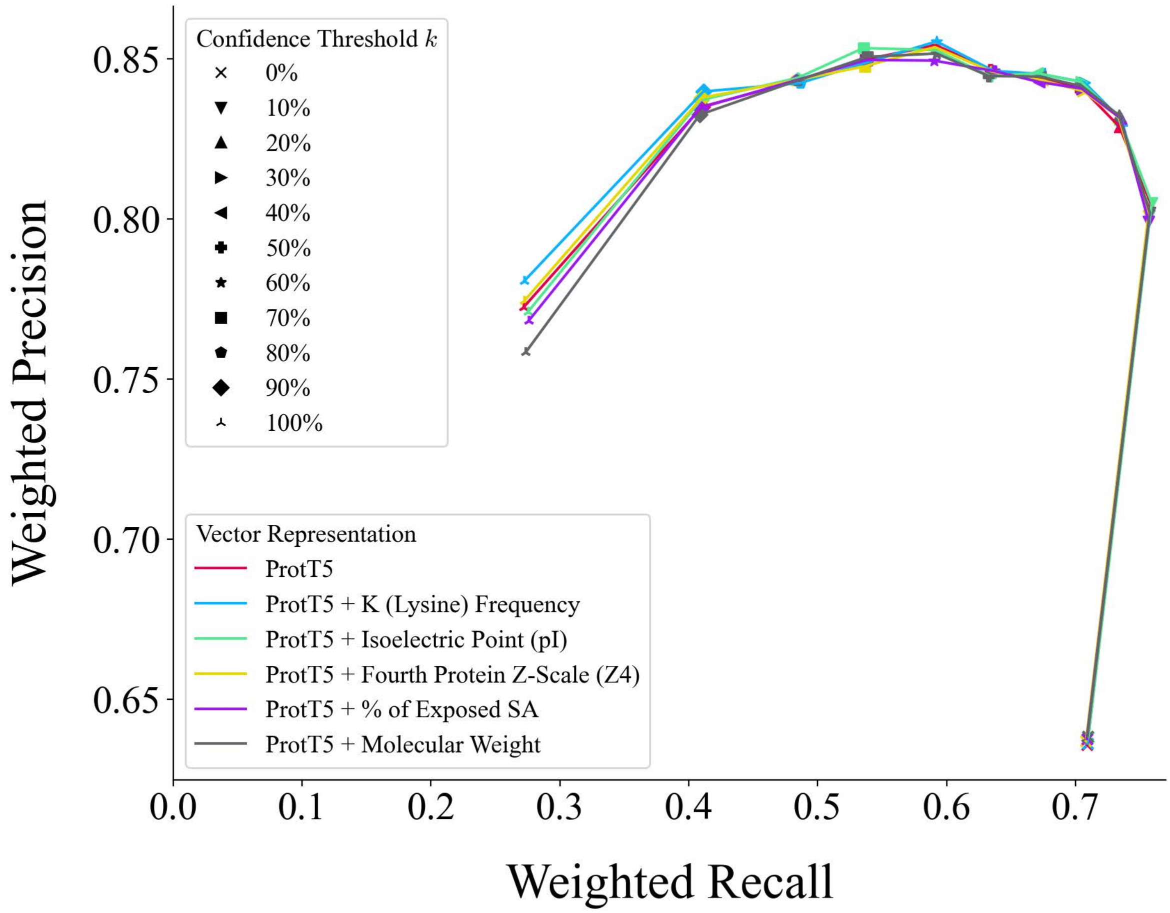

S3 Figure. Weighted precision-recall curves after integrating handcrafted protein sequence properties to the vector representations of the RBPs. The selected protein sequence properties are those with the highest Gini importance after training the phage-host interaction prediction tool by Boeckaerts *et al.* [15] on our dataset. Note that the fourth protein Z-scale (Z4) [60] is related to the heat of formation, hardness, electronegativity, and electrophilicity; *% of Exposed SA* refers to the percentage of residues with exposed solvent accessibility. The curves plot the weighted precision against the weighted recall at different confidence thresholds ranging from $k = 0\%$ to 100% in steps of 10%.

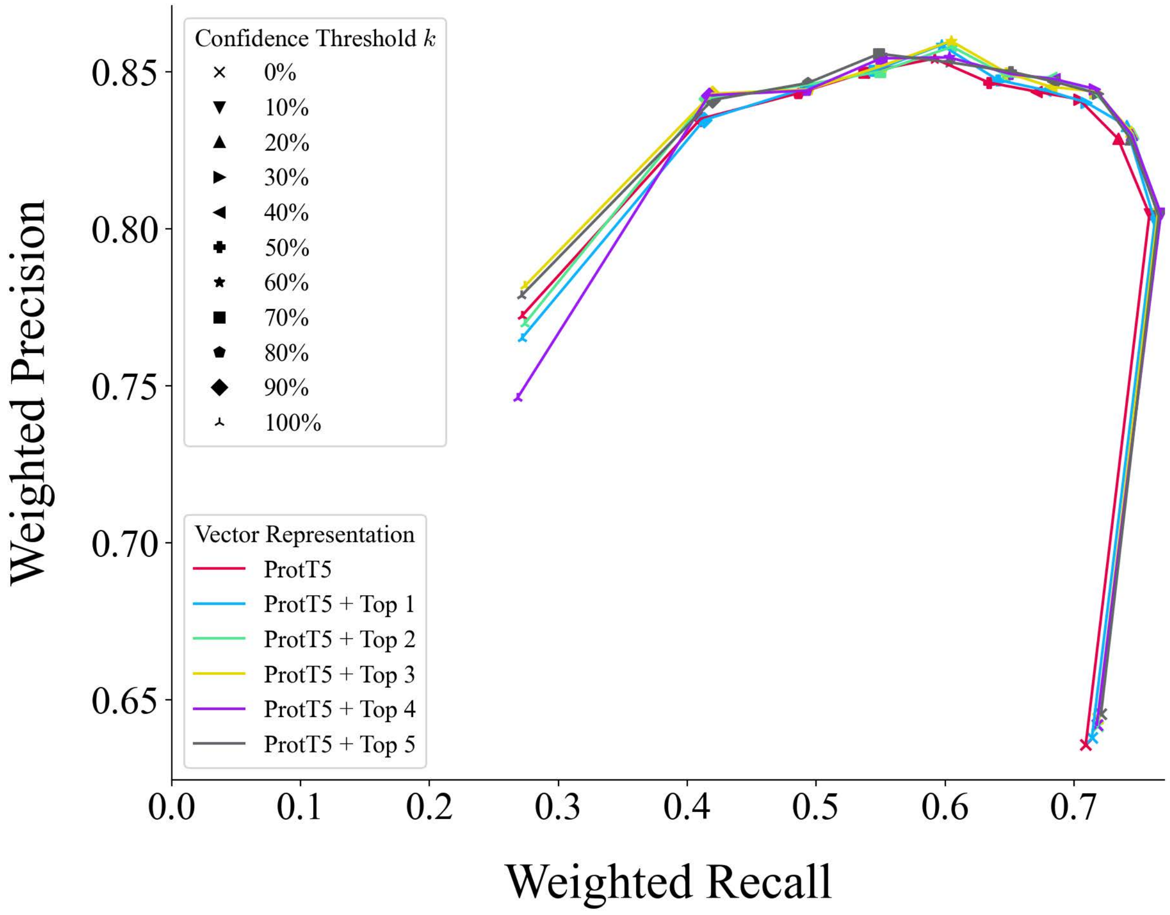

S4 Figure. Weighted precision-recall curves after integrating the top n handcrafted sequence properties to the vector representations of the RBPs. The selected sequence properties are those with the highest Gini importance after training the phage-host interaction prediction tool by Boeckaerts *et al.* [15] on our dataset. These properties (in order of decreasing importance) are the A nucleotide frequency, GC content, C nucleotide frequency, TTA codon frequency, and TTA codon usage bias. The curves plot the weighted precision against the weighted recall at different confidence thresholds ranging from $k = 0\%$ to 100% in steps of 10%.

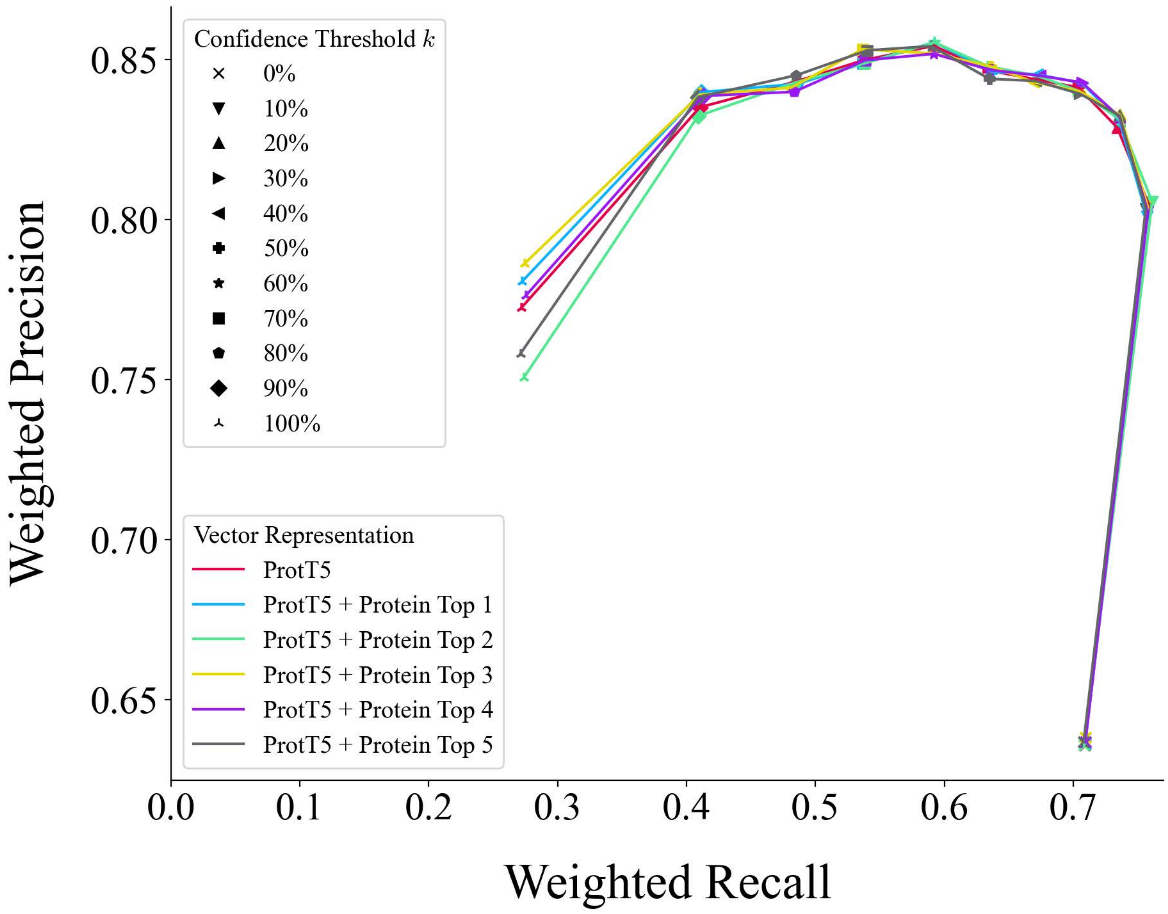

S5 Figure. Weighted precision-recall curves after integrating the top n handcrafted protein sequence properties to the vector representations of the RBPs. The selected protein sequence properties are those with the highest Gini importance after training the phage-host interaction prediction tool by Boeckaerts *et al.* [15] on our dataset. These properties (in order of decreasing importance) are the K (lysine) frequency, isoelectric point, fourth protein Z-scale [60] (which is related to the heat of formation, hardness, electronegativity, and electrophilicity), percentage of residues with exposed solvent accessibility, and molecular weight. The curves plot the weighted precision against the weighted recall at different confidence thresholds ranging from $k = 0\%$ to 100% in steps of 10%.
